# The geographic distribution, and the biotic and abiotic predictors of select zoonotic pathogen detections in Canadian polar bears

**DOI:** 10.1038/s41598-024-62800-x

**Published:** 2024-05-26

**Authors:** Christina M. Tschritter, Peter van Coeverden de Groot, Marsha Branigan, Markus Dyck, Zhengxin Sun, Emily Jenkins, Kayla Buhler, Stephen C. Lougheed

**Affiliations:** 1 Department of Biology, Queen’s University, Kingston, ON Canada; 2 Department of Environment and Climate Change, Government of the Northwest Territories, Inuvik, Northwest Territories Canada; 3 Department of Environment, Government of Nunavut, Igloolik, NT Canada; 4 Western College of Veterinary Medicine (WCVM), Saskatoon, SK Canada

**Keywords:** Pathogens, Microbial genetics

## Abstract

Increasing Arctic temperatures are facilitating the northward expansion of more southerly hosts, vectors, and pathogens, exposing naïve populations to pathogens not typical at northern latitudes. To understand such rapidly changing host–pathogen dynamics, we need sensitive and robust surveillance tools. Here, we use a novel multiplexed magnetic-capture and droplet digital PCR (ddPCR) tool to assess a sentinel Arctic species, the polar bear (*Ursus maritimus*; n = 68), for the presence of five zoonotic pathogens (*Erysipelothrix rhusiopathiae, Francisella tularensis, Mycobacterium tuberculosis* complex, *Toxoplasma gondii* and *Trichinella* spp.), and observe associations between pathogen presence and biotic and abiotic predictors. We made two novel detections: the first detection of a *Mycobacterium tuberculosis* complex member in Arctic wildlife and the first of *E. rhusiopathiae* in a polar bear. We found a prevalence of 37% for *E. rhusiopathiae*, 16% for *F. tularensis*, 29% for *Mycobacterium tuberculosis* complex, 18% for *T. gondii*, and 75% for *Trichinella* spp. We also identify associations with bear age (*Trichinella* spp.), harvest season (*F. tularensis* and MTBC), and human settlements (*E. rhusiopathiae, F. tularensis*, MTBC, and *Trichinella* spp.). We demonstrate that monitoring a sentinel species, the polar bear, could be a powerful tool in disease surveillance and highlight the need to better characterize pathogen distributions and diversity in the Arctic.

## Introduction

In Arctic ecosystems, where climate change is disproportionately impactful, we expect to see large changes in the regional diversity, intensity, and epidemiology of infectious diseases^1,2^. Increasing temperatures and humidity is predicted to facilitate the northward expansion of more southerly hosts, vectors, and pathogens, exposing naïve hosts to pathogens not common at northern latitudes^3,4^. Additionally, Arctic pathogens may experience changes in life cycle phenology and environmental persistence, ultimately affecting their prevalence and levels of morbidity^5^. The impact of such pathogens may extend beyond the Arctic, mediated by the infection of migratory host species^6,7^ (e.g., birds, ungulates, marine mammals) and increasing human traffic through Arctic regions^8^. To better understand and potentially mitigate the effects of novel and emerging pathogens, we need more information on existing geographic distributions and how changes in climate and other anthropogenic stressors may impact them. Monitoring vast and remote regions like the Arctic is economically and logistically challenging; thus, harvest-based monitoring of pathogen occurrences in sentinel species may offer a cost-effective framework for disease surveillance^9^.

Apex predators, like the polar bear (*Ursus maritimus*), are sentinel species of environmental change that can serve as indicators for shifts in the prevalence of pathogens within food webs^10^. The vast home ranges of polar bears (up to > 350,000 km^2^), encompassing both terrestrial and marine ecosystems, increase the likelihood of pathogen exposure and make them excellent proxies for pathogen presence across large geographic scales^10^. Thus, monitoring pathogen presence in polar bear populations may allow inferences on the distribution of specific pathogens and potentially highlight areas of high prevalence and range expansion.

For purposes of management, the polar bear range is divided into 19 management units, 13 of which fall partially or wholly in Canada^11^. The biological relevance of management units is unclear and other means of classifying polar bears populations have been proposed, including genetically distinct regional clusters^12–15^ and the delimitation of ice ecoregions (Table 1). Ice ecoregions are based on differences in observed patterns of ice formation, movement, and melt, and how polar bears respond to those patterns^16^. This is increasingly biologically relevant as regional diminution of multi-year sea ice is concentrating bears on land, increasing population densities, and extending the duration of the nutritionally stressful fasting period. The concurrence of immunological stressors and arrival of new pathogens may provide opportunistic pathogens with means for novel infection and transmission^17^. High throughput screening tools can enhance disease surveillance across the Arctic and provide high-resolution data to delineate focal pathogen distributions, detect novel occurrences, and assess the relationships between pathogen incidence and geographical and biological factors that may facilitate their spread.

**Table 1:** Sample distribution by ice ecoregion and sample type, with *n* referring to the number of individuals, and *n* muscle and *n* liver referring to the number of samples of each tissue type, as well as the polar management units.

Ice ecoregion	*n*	*n* muscle	*n* liver	Total samples	Canadian polar bear management unit(s) within ice ecoregion
Divergent ice ecoregion	4	0	4	4	Southern Beaufort Sea
Convergent ice ecoregion	15	4	15	19	Northern Beaufort Sea
Archipelago ice ecoregion	14	13	14	27	Norwegian Bay, Viscount Melville Sound, Lancaster Sound, Gulf of Boothia, Kane Basin, and Mc'Clintock Channel
Seasonal ice ecoregion	35	33	31	64	Foxe Basin, Western Hudson Bay, Southern Hudson Bay, Baffin Bay, and Davis Strait
Total	68	50	64	114	

### Pathogen selection rationale

We focus on five pathogens based on documented presence in Arctic ecosystems, known or potential impacts on human health, and relevance to the health of domestic animals and wildlife. *Erysipelothrix rhusiopathiae* is increasingly detected in Arctic wildlife^18–20^ and peoples^21^. For example, the muskox (*Ovibos moschatus*) population in the Western Canadian Arctic has declined by ~ 50% since the early 2000’s^22^, with recent die-offs associated with *E. rhusiopathiae*^19,23^. By scavenging and/or hunting infected animals, bears could be exposed and, because inoculated carnivores can shed bacteria in their feces, they have the potential to facilitate bacterial spread in the environment^24^.

*Francisella tularensis* is a zoonotic bacterium that can cause the disease tularemia. It is commonly divided into two subspecies, the most common of which in the circumpolar North is the less pathogenic subspecies *F. tularensis holarctica*. The more virulent Type A is endemic to North America and classified as a Category A potential bio threat agent by the Centers for Disease Control, USA (CDC)^25,26^. The bacterium has two primary disease cycles: Type A occurs predominantly in terrestrial environments, where transmission occurs through vectors like ticks, mosquitoes, and biting flies^27^ with lagomorphs and rodents acting as reservoirs^28,29^; Type B occurs in aquatic environments, where the bacteria is ingested via a contaminated water-source^30,31^. Seroprevalence in polar bears has increased with variation across the polar bear range (Beaufort Sea 5% vs. Western Hudson Bay > 60%)^32,33^. Without treatment humans infected with *F. tularensis* (Type A) have a high mortality rate^34^; however, seroprevalence in polar bear and Arctic fox indicates non-fatal exposure may be common in Arctic wildlife.

The *Mycobacterium tuberculosis* complex (MTBC; *M. tuberculosis*, *M. bovis*, *M. bovis* BCG, *M. africanum*, *M. microti*, *M. mungi*, *M. canetti*, *M. caprae*, *M. pinnipedii,* and *M. orygis*) is a group of bacteria that can cause tuberculosis in both animals and people^35^. MTBC members have been documented in > 40 wildlife species, and in 2018 > 150,000 human tuberculosis cases globally were reported to be of zoonotic origin, mostly *M. bovis* from cattle and ungulates^35^. In 2020, Canada reported a 15-fold increase in the prevalence of tuberculosis (primarily *M. tuberculosis* Euro-American lineage 4.8) between the Inuit and the general population^36^. This high incidence led the Inuit Tapiriit Kanatami (the representative organization for Inuit in Canada) and the federal government to prioritize the elimination of tuberculosis from Inuit regions by 2030^37^. The zoonotic nature of MTBC, the high morbidity, and the relevance of *M. tuberculosis* to the health of Arctic peoples suggests that surveillance for wildlife reservoirs is paramount^35,38^.

We included two zoonotic parasites: *Toxoplasma gondii* (an apicomplexan protozoan) and *Trichinella* spp. (muscle-dwelling nematodes), both previously observed in polar bears^32,33,39^. These zoonotic parasites are transmitted among hosts through the consumption of undercooked or raw meat by carnivory or scavenging^40^. *Toxoplasma gondii* can cross the blood–brain barrier and the placenta in mammals^39,41^, resulting in birth defects and miscarriages^42^. Thus, *T. gondii* is a concern in the Arctic where regional estimates of human seroprevalence range from 8 to 60% and congenital outbreaks have been reported^43–46^. Outbreaks of human trichinellosis in Canada are usually due to the consumption of bear or walrus meat prepared to an insufficient temperature to inactivate the species of this parasite endemic in the Canadian North (*Trichinella nativa* and *Trichinella T6*)*.* Specifically, in Indigenous communities, where sharing harvested food is culturally significant, this can result in trichinosis outbreaks. The largest documented case in Canada involved 78 individuals who shared black bear meat in Northern Saskatchewan^47^.

Here, we survey these five zoonotic pathogens in polar bears from across the Canadian Arctic using a novel multiplexed magnetic-capture and ddPCR tool^9^. We first map the geographical distribution of detections for each pathogen across the Canadian Arctic. Based on previous work, we expect to see non-uniform distributions of pathogens among regions, between sexes and among age classes, driven by behavioural (male vs female, juvenile vs sexually mature) and ecological (sea ice dynamics) differences. Further, large human settlements may occur near more biologically productive regions (e.g., in proximity to good fishing, hunting and fresh water), and produce waste, which can attract bears. An extension of this is that subadults or more nutritionally stressed bears may be more likely to seek supplementary food sources such as refuse in dumps, specifically during periods of fasting (i.e., the ice-free period)^48^. Thus, we test for associations between pathogen presence and seasonality, proximity to human settlement, and waste accumulation (m^3^) at landfills in the nearest settlement (Table 2).

**Table 2:** A summary of the biological and geographical predictor variables used in the univariate and recursive partitioning analyses, their descriptions, and their sources if applicable.

Predictor variable	Data type	*n*	*n* muscle	*n* liver	Description
*Biological*					
Sex	(0/1)	M = 46; F = 22	M = 36; F = 14	M = 43; F = 21	Sex as reported by hunter/ harvester
Age	(0/1)	Subadult = 28; Adult = 36	Subadult = 19; Adult = 29	Subadult = 26; Adult = 34	Age class was assigned based on estimations provided by the hunters: ‘subadult’ (between 2 and 5 years of age) and ‘adult’ (≥ 6 years of age; Derocher & Stirling, 1996; Lunn & Stirling, 1985)
*Geographic*					
Season	(0/1)	Summer = 13; Winter = 55	Summer = 13; Winter = 37	Summer = 12; Winter = 52	Seasonality was assessed by first dividing the Arctic year into two seasons ‘summer’ and ‘winter’, the summer season being defined as between June 19 and November 19 for the Hudson Bay region and from June 25–October 15 for the remainder of the Canadian arctic (*Canadian Environmental Sustainability Indicators: Sea ice in Canada*, 2021)
Distance to nearest settlement (km)	Continuous	2.6–322.3 km	2.6–322.3 km	2.6–322.3 km	Distance to the nearest Arctic settlement is calculated based on the least distance between the bears harvest location and the GPS locations and census data provided by Statistics Canada 2016 Census data
Waste accumulation in nearest settlement (m^3^)	Continuous	13,125–564,625 m^3^	13,125–564,625 m^3^	13,125–564,625 m^3^	Waste accumulation (m^3^) estimates were obtained for each settlement from publicly sourced data on the status of waste disposal infrastructure across the Canadian Arctic (Towards a waste-free Arctic, 2021)

## Results

### Digital droplet PCR detections

**Figure 1:**
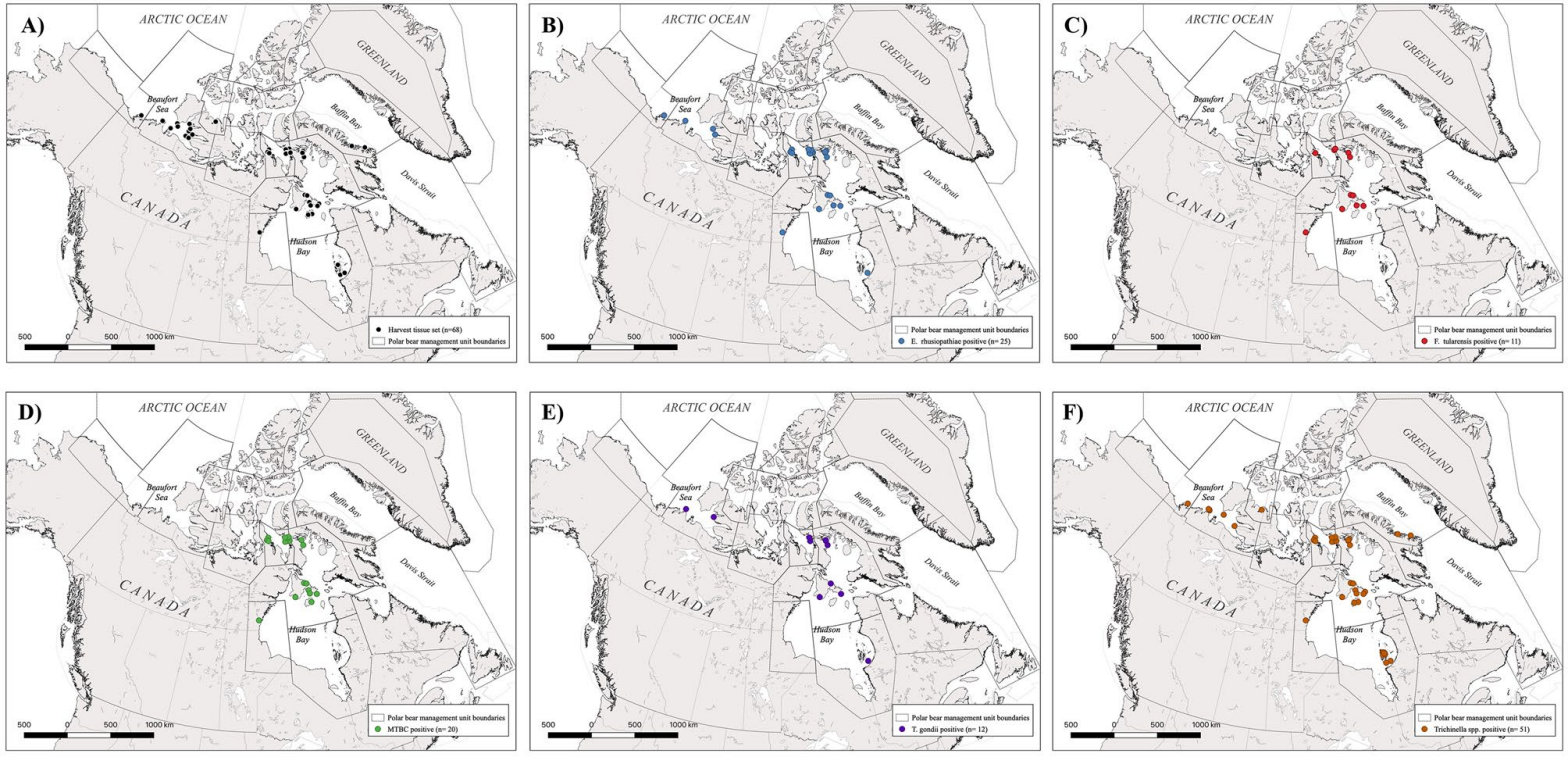
The harvest location of (**A)** all harvest tissue sets included in the analyses (n = 68), and the positive detections for (**B)** *E. rhusiopathiae* (n = 25), (**C)** *F. tularensis* (n = 11), (**D)** *Mycobacterium tuberculosis* complex (MTBC; n = 20), (**E)** *T. gondii* (n = 12), and (**F)** *Trichinella* spp. (n = 51). All maps are overlayed with the boundaries of the polar management units that fall within Canada. These maps were generated using free and open source QGIS software (*v*3.30.3; https://qgis.org).

**Figure 2:**
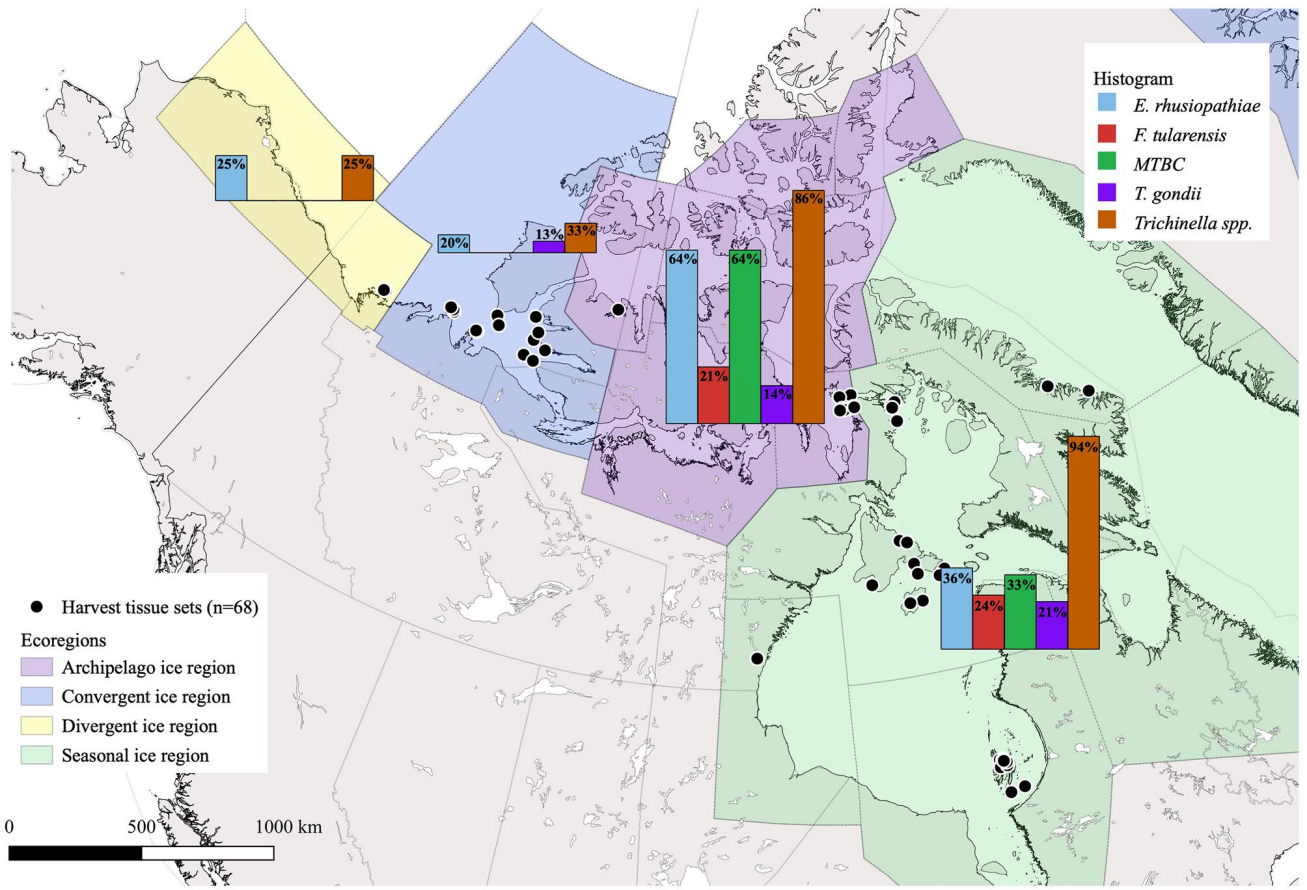
Prevalence estimates of the five focal pathogens (*E. rhusiopathiae*, *F. tularensis*, MTBC, *T. gondii,* and *Trichinella* spp.) based on individual status, per ice ecoregion (Divergent, Convergent, Archipelago, and Seasonal ice ecoregions), as described by Amstrup et al., (2007). The average standard error of the mean difference of prevalence is 52.57, 31.26, 36.17, and 27.04 for the Divergent, Convergent, Archipelago, and Seasonal ice ecoregions, respectively. This map was generated using free and open source QGIS software (*v*3.30.3; https://qgis.org).

Of the samples assessed (n = 114; 64 liver and 50 muscle; n = 68 individuals), the triplex assay detected 34 tissue samples positive for *Erysipelothrix rhusiopathiae* (originating from 25 unique individuals, 37% prevalence), 16 positive tissues for *Francisella tularensis* (originating from 11 individuals; 16% prevalence), and 34 tissue positives for MTBC (originating from 20 individuals; 29% prevalence). The identification of an MTBC member was confirmed through additional testing for the IS6110 and 16S genes specific to the MTBC and the *Mycobacterium* spp. respectively (Tschritter and Lougheed unpublished data 2023). The duplex assay detected 12 tissues positive for *Toxoplasma gondii* (representing 12 individuals;18% prevalence) and 76 positives for *Trichinella* spp. (originating from 51 unique individuals; 75% prevalence). The data are summarized in Figs. 1 and 2.

### Prevalence estimates by ice ecoregion

The full results of the prevalence estimates are reported in Table 3 while a summary (based on individual status) is depicted in Fig. 2. In the Divergent ice ecoregion (western Arctic), we found 25% prevalence (individuals) for both *E. rhusiopathiae* and *Trichinella* spp. Similarly, we found an individual prevalence of 20% , 13% , and 33% (for *E. rhusiopathiae, T. gondii,* and *Trichinella* spp. respectively, in the Convergent ice ecoregion (west–central Arctic). In the Archipelago ice ecoregion (central Arctic), we found an individual prevalence of 64% for *E. rhusiopathiae*, 21% for *F. tularensis*, 64% for MTBC, 14% for *T. gondii*, and 86% for *Trichinella* spp. Finally, in the Seasonal ice ecoregion (south-central Arctic), we observed an individual prevalence of 34% for *E. rhusiopathiae*, 23% for *F. tularensis*, 31% for MTBC, 23% for *T. gondii*, and 94% for *Trichinella* spp. We observed an average (across the five pathogens) standard error of the mean difference of 52.57 for the Divergent, 31.26 for the Convergent, 36.17 for the Archipelago, and 27.04 for the Seasonal ice ecoregions. These high values reflect the relatively small sample sizes for each ice ecoregion.

**Table 3:** A summary of the pathogen prevalence estimates by ice ecoregion, the standard error of the mean difference, and the 95% confidence intervals calculated based on the Wilson Score Interval (recommended for small sample sizes; Brown et al., 2001).

	Divergent	Convergent	Archipelago	Seasonal
*E. rhusiopathiae*				
*n*	25% [57.18; 4.56–69.93]	20% [34.95; 7.05–45.18]	64% [38.2; 38.76–83.65]	34% [29.15; 20.83–50.85]
*n* muscle	NA	50% [59.16; 15–85]	54% [39.68; 29.14–76.79]	33% [29.54; 19.75–50.39]
*n* liver	25% [57.18; 4.56–69.94]	20% [34.96; 7.05–45.2]	21% [36.07; 7.57–47.59]	23% [28.54; 11.4–39.81]
*F. tularensis*				
*n*	0% [49.5; 0–48.99]	0% [25.56; 0–20.39]	21% [36.07; 7.57–47.59]	23% [27.62; 12.06–39.02]
*n* muscle	NA	0% [49.5; 0–48.99]	23% [37.29; 8.18–50.26]	21% [27.77; 10.68–37.75]
*n* liver	0% [49.5; 0–48.99]	0% [25.56; 0–20.39]	0% [26.46; 0–21.53]	19% [27.87; 9.19–36.28]
*MTBC*				
*n*	0% [49.5; 0–48.98]	0% [25.56; 0–20.38]	64% [38.2; 38.76–83.65]	31% [28.86; 18.55–47.98]
*n* muscle	NA	0% [49.5; 0–48.99]	62% [39.31; 35.52–82.29]	33% [29.54; 19.75–50.39]
*n* liver	0% [49.5; 0–48.99]	0% [25.56; 0–20.39]	50% [38.84; 26.8–73.2]	26% [29.54; 19.75–50.39]
*T. gondii*				
*n*	0% [49.5; 0–48.99]	13% [33.08; 3.74–37.88]	14% [34.18; 4.01–39.94]	23% [27.62; 12.06–39.02]
*n* muscle	NA	50% [59.16; 15–85]	8% [32.49; 1.37–33.31]	24% [28.34; 12.83–41.02]
*n* liver	0% [49.5; 0–48.99]	0% [25.56; 0–20.39]	7% [31.33; 1.3–31.47]	0% [17.78; 0–11.03]
*Trichinella* spp.				
*n*	25% [57.18; 4.56–69.94]	33% [37.17; 15.18–58.29]	86% [34.18; 60.06–96]	94% [21.95; 81.39–98.42]
*n* muscle	NA	50% [59.16; 15–85]	85% [35.39; 57.76–95.67]	94% [22.6; 80.39–98.32]
*n* liver	25% [57.18; 4.56–69.94]	27% [36.27; 10.9–51.95]	64% [38.2; 38.76–83.66]	58% [30.67; 40.77–73.58]

### Univariate analyses

The results of the univariate analyses are summarized in Table 4. We identified an association between *E. rhusiopathiae* detection and increasing waste accumulation in the nearest settlement (m^3^). For both *F. tularensis* and MTBC detections we found significant relationships with season of harvest, with the pathogens detected more frequently in the summer, and increasing waste accumulation in the nearest settlement (m^3^). *Trichinella* spp. detection increased significantly with bear age, and waste accumulation (m^3^), and was more commonly detected in muscle tissue of male vs female bears. We found no significant associations for *T. gondii*.

**Table 4:** A summary of the univariate statistical analyses and the results by individual (detections in either or both tissues) and each tissue type (muscle and liver). Significant values and predictors are bolded for clarity.

Predictor variable	Statistical test	Individual (P-value)	Muscle (P-value)	Liver (P-value)
*E. rhusiopathiae*				
Sex	Fisher’s exact test	0.6	1	0.36
Age	Fisher’s exact test	1	1	0.33
Season	Fisher’s exact test	0.056	0.33	0.44
Distance to nearest settlement (km)	Logistic regression	0.67	0.65	0.53
**Waste accumulation in nearest settlement (m**^**3**^**)**	Logistic regression	**0.01**	0.39	0.57
*F. tularensis*				
Sex	Fisher’s exact test	0.74	1	1
Age	Fisher’s exact test	1	1	1
**Season**	Fisher’s exact test	**0.0043**	0.1	**0.0093**
Distance to nearest settlement (km)	Logistic regression	0.93	0.55	0.75
**Waste accumulation in nearest settlement (m**^**3**^**)**	Logistic regression	**0.025**	0.35	0.49
*MTBC*				
Sex	Fisher’s exact test	0.57	1	0.75
Age	Fisher’s exact test	1	1	0.55
**Season**	Fisher’s exact test	**0.0011**	**0.017**	**0.025**
Distance to nearest settlement (km)	Logistic regression	0.18	0.19	0.49
**Waste accumulation in nearest settlement (m**^**3**^**)**	Logistic regression	**0.0069**	0.18	**0.034**
*T. gondii*				
Sex	Fisher’s exact test	0.74	1	1
Age	Fisher’s exact test	0.74	1	1
Season	Fisher’s exact test	0.22	0.44	1
Distance to nearest settlement (km)	Logistic regression	0.81	0.86	0.16
Waste accumulation in nearest settlement (m^3^)	Logistic regression	0.27	0.56	0.2
*Trichinella* spp.				
**Sex**	Fisher’s exact test	0.77	**0.044**	0.29
**Age**	Fisher’s exact test	**0.016**	**0.03**	0.6
Season	Fisher’s exact test	0.16	1	0.11
Distance to nearest settlement (km)	Logistic regression	0.4	0.77	0.12
**Waste accumulation in nearest settlement (m**^**3**^**)**	Logistic regression	**0.015**	0.97	0.15

### Recursive partitioning of variables

**Figure 3:**
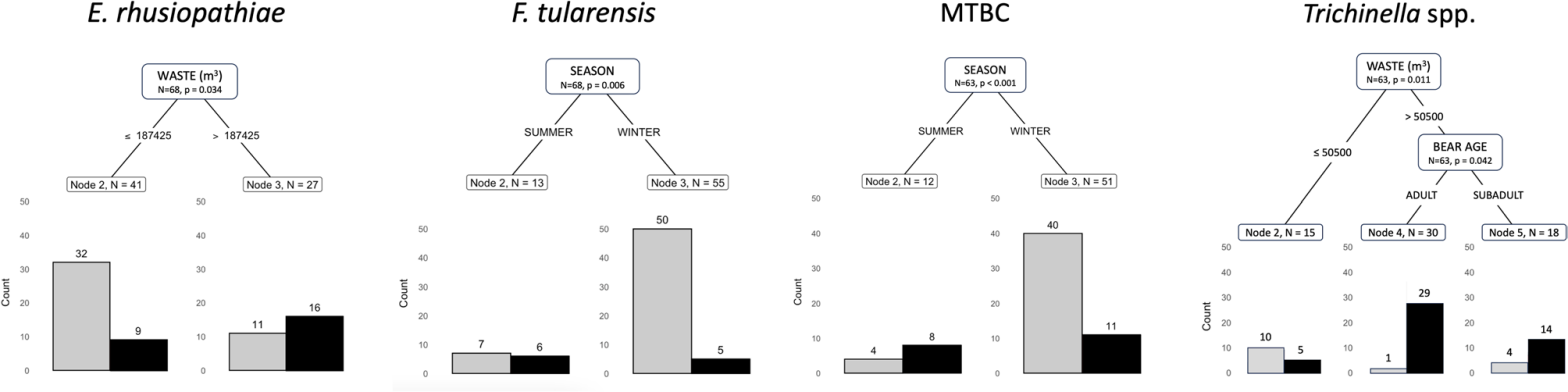
The conditional inference trees (*partykit*^89^; E*. rhusiopathiae* and *F. tularensis*) and the generalized linear mixed-model decision trees (*glmertree*^88^; MTBC and *Trichinella* spp.) resulting from the recursive partitioning analyses, the grey bars representing a negative detection and the black bars representing a positive detection for the respective pathogens.

The relevant decision trees are presented in Fig. 3. The variables of ‘tissue type’, ‘harvest year’, and ‘ecoregion’ had no effect on the occurrence of *E. rhusiopathiae* and *F. tularensis*, and therefore the random effects were excluded and conditional inference trees were generated. ‘Harvest year’ and ‘ecoregion’ had ICC values of 0.928 and 0.768 respectively for MTBC detection. ‘Tissue type’ had an ICC value of 0.727 for *T. gondii* and ‘harvest year’ an ICC value of 0.542 for *Trichinella* spp. detection. Thus, mixed-model decision trees were generated for MTBC (RE: harvest year and ecoregion), *T. gondii* (RE: tissue type), and *Trichinella* spp. (RE: harvest year). Increased waste accumulation (m^3^) in the nearest settlement was a top predictor for *E. rhusiopathiae* (*p* = 0.034), with detection more likely in bears nearest to settlements with > 187,425 m^3^ of waste. Harvest during the summer season was a top predictor for *F. tularensis* (*p* = 0.006) and MTBC (*p* < 0.001) detection. *Trichinella* spp. detection was associated with increased waste accumulation in the nearest settlement and bear age, with adulthood being an important predictor in bears nearest to settlements with > 50,500 m^3^ of waste. No significant predictors were identified for *T. gondii*.

### Co-occurrence

We found that two relationships held across the tissue-specific datasets: *E. rhusiopathiae* and *F. tularensis* (individual *p* = 0.0013, muscle *p* < 0.001, and liver *p* = 0.0014), and MTBC (individual *p* < 0.001, muscle *p* < 0.001, liver *p* = 0.002) and *F. tularensis* (Supplementary material). In both cases, the pathogens occurred together more frequently than would be expected by chance; this observation held across the metrics of co-occurrence (Jaccard’s index, Sørensen-Dice index, Simpson Index, and the affinity metric of co-occurrence; Supplementary material). We found that an individual positive for *E. rhusiopathiae* was 7 times more likely to be positive for *F. tularensis* than individuals where *E. rhusiopathiae* was not detected*,* with 9/11 individuals positive for *F. tularensis* also testing positive for *E. rhusiopathiae* (OR = 7; 95% CI: 2, 43; *p* = 0.0013). An individual positive for MTBC detection was 21 times more likely to be positive for *F. tularensis*, with 10/11 individuals positive for *F. tularensis* also testing positive for MTBC (OR = 21; 95% CI: 5, 199; *p* < 0.001). Only 9 of the 25 individuals positive for *E. rhusiopathiae* and 10 of the 20 MTBC positive individuals also tested positive for *F. tularensis*.

## Discussion

Our study is the first to use multiplexed ddPCR data to map the distributions of zoonotic pathogens in a wildlife species in the Canadian Arctic. We found the highest occurrences of target pathogens in the central and south-central Arctic (Archipelago and Seasonal ice ecoregions; Fig. 2). The factors associated with pathogen presence varied, with bear age (*Trichinella* spp.), harvest season (*F. tularensis* and MTBC), and human settlements (*E. rhusiopathiae*, *F. tularensis*, MTBC, and *Trichinella* spp.) found to be important factors. Our study, while preliminary, illustrates how different ecological and geographical factors may shape the distribution of pathogens, but also showcases the power of molecular detection tools and broad monitoring enabled by collaborations with communities and northern governments. We explore these themes in more detail below.

For the first time, we report the detection of a *Mycobacterium tuberculosis* complex member in any Arctic wildlife, the first detection of *E. rhusiopathiae* in a polar bear, and the first molecular detection of *F. tularensis* in the Arctic tundra with previous detections being serological^49^. Given the low concentrations of pathogen DNA identified and limited sample type and quality, more research is required to definitively establish which MTBC member is present, the subspecies of *F. tularensis* and to explore the implications of these discoveries for Arctic peoples and wildlife. The observations of MTBC and *E. rhusiopathiae* DNA do not imply that these bacteria are new to the Arctic or to polar bears, but rather that previous studies have lacked the scope, geographic sampling intensity, and/or molecular tools to detect these pathogens in small amounts of host tissue. Further work is needed to determine if polar bears are infected with live bacteria, the health significance for polar bears and peoples, and whether bears have adapted to the presence of these pathogens, acquiring some level of host-immunity.

Bears harvested during the summer season were more likely to test positive for *F. tularensis* and MTBC (Supplementary material). The Arctic summer offers increased chances of polar bear exposure to terrestrial bacterial pathogens because of seasonal changes in bear ecology, concurrent with a shift to a more moderate climate. Milder summer temperatures enable longer persistence of pathogens in feces and contaminated food or water sources and increase vector species activity and abundance^7^. The seasonal absence (or reduction) of sea-ice cover forces bears to concentrate on land, where they fast for the duration of the season, decreasing immune function^17^. Further, increased scavenging behaviours on land may increase bear exposure to potential reservoir populations like rodents or waterfowl, and also increase foraging on shared food sources, like whale carcasses^33,50^. Regional differences in bear migration ecology, driven by sea-ice dynamics, may shape pathogen distributions. For example, Western Hudson Bay bears spend more time on land during the summer season relative to the Beaufort Sea bears, and this may be a primary cause of geographic differences in seroprevalence of *F. tularensis*^32,33^. Our data support the observation of lower prevalence of *F. tularensis* and MTBC in the western Arctic, potentially caused by these differences in migration patterns.

We found that greater waste accumulation (m^3^) in the nearest settlement was positively associated with detections of *E. rhusiopathiae*, F*. tularensis*, MTBC, and *Trichinella* spp. (Supplementary material). Increased waste in settlements can attract bears that may be seeking supplementary food, which is particularly relevant during periods of nutritional stress (e.g., ice-free period, gestation) and for bears that are less adept at hunting (subadults, unhealthy animals, and family groups)^48^. Additionally, the attraction of supplementary food may draw bears farther inland than they would otherwise travel, increasing their exposure to biting insects and other vector or reservoir populations. Scavenging at waste disposal sites can increase exposure to terrestrial pathogens through organic waste (e.g., carcasses of human-hunted or trapped wildlife, food waste, etc.) and facilitate the land-to-sea movement of these pathogens. This is supported by previous studies, which have identified “capture in human settlement” as a predictor of *Trichinella* seroprevalence^32^. This implies that bears living close to human settlements may either be at greater risk of exposure to *Trichinella* spp. (possibly through the scavenging of discarded carcasses) and/or that bears with higher exposure to *Trichinella* spp. may be marginal and more likely to seek supplementary food in or near human settlements.

We detected *E. rhusiopathiae* in 37% of polar bears with a wide geographic distribution (Fig. 1B and Fig. 2) and found an association with increasing waste accumulation (m^3^) in the nearest settlement (Supplementary material). The bacterium is typically transmitted through ingestion of contaminated food and water but can also survive in the soil and decomposing matter for extended periods^51,52^. Deaths of muskox in the Canadian Arctic Archipelago (2012) were attributed to *E. rhusiopathiae* with the suggestion that the pathogen may persist in the Arctic because of non-ungulate host species, including rodents and migratory waterbirds^19,23^. Our results identify the polar bear as a potential host species for *E. rhusiopathiae* and recent investigations into the species’ role in the Arctic ecosystem parallel our findings by suggesting that it is more prevalent than previously understood^53,54^. Multiple factors have been posited to play a role in the distribution of *E. rhusiopathiae,* including climate and seasonality (e.g., seasonal increases in seropositivity in tundra caribou^52^; multiple stressors implicated in *E. rhusiopathiae*^53^). Biting insects may serve as a vector for *E. rhusiopathiae*^55^ and may explain coincidence with *F. tularensis*, known to be carried by insect vectors. Similar to *Trichinella*, we postulate that the relationship identified with waste accumulation (m^3^) may be a product of scavenging behaviours targeting the readily available carcasses of hunter harvested ungulates. While previous work has emphasized terrestrial hosts, *E. rhusiopathiae* may originate in marine ecosystems, causing severe and fatal disease in captive^23,56,57^ and wild cetaceans^54,58^ and other marine species^59^. Given the ability of polar bears to move between marine and terrestrial ecosystems, we posit that they could facilitate the sea-to-land transfer of pathogens from infected marine species.

We found 16% of individuals positive for *F. tularensis*, with the highest prevalence in the central and south-central (21–24%; Archipelago and Seasonal ice ecoregion; Fig. 1C) Arctic regions. Positive detections were higher in summer and related to waste accumulation (m^3^) in the nearest community. Seroprevalence in Arctic fox and polar bears is elevated in the central Arctic relative to the western Arctic^32,33,49^; though recent seroprevalence data from Alaskan polar bears suggest that *F. tularensis* is increasing in the western Arctic^60^. Rodent species are hypothesized to be primary reservoirs for several bacterial pathogens, including *F. tularensis*^32,49^ and *E. rhusiopathiae*^52^. Lemming (*Dicrostonyx* spp., *Lemmus trimucronatus*) and vole (*Clethrionomys rutilus*, *Microtus* spp., and *Myodes gappi*) species experience an increase in population densities during winter, coinciding with occupancy of the subnivean hibernacula that is more conducive to pathogen persistence^61,62^. Buhler et al. (2022) documented a 2018 outbreak of tularemia in the Arctic fox population following a peak in vole abundance and posited that more aquatic voles (vs lemmings) play an important role in the maintenance and transmission of *F. tularensis* in inland Arctic ecosystems. We found the highest prevalence of the focal bacterial pathogens outside of the reported vole ranges and thus, voles may not serve as the primary reservoir for these bacteria (Supplementary material).

We detected MTBC member(s) DNA in 29% of individuals assessed, with the highest prevalence in the central Arctic (64%; Archipelago ice ecoregion; Fig. 1D). We also found an association with the summer season and waste accumulation (m^3^) in the nearest settlement (Supplementary material). Despite this being the first reported detection of an MTBC in Arctic wildlife, there has been speculation that *Mycobacterium pinnipedii* could be present in Arctic seal populations^63,64^. Until now, the most northern confirmed occurrence of an MTBC member in wildlife is *M. bovis* in the wood bison (*Bison bison athabascae*) of Wood Buffalo National Park (59.5°N)^65,66^. Human contamination of our samples is unlikely given that samples were derived from multiple communities, that multiple harvesters contributed samples within communities, and that the likelihood of aerosol transmission onto sample surface is low. It is also possible that this is a novel, previously undescribed species of MTBC that has evolved from *M. tuberculosis*; for example, *M. mungi* was newly characterized in 2010, originating from banded mongoose (*Mungos mungo*) populations living in close proximity to and scavenging waste from humans with high incidence of *M. tuberculosiss*^67^*.* Despite an association between MTBC detection and waste accumulation (m^3^), the low annual average of 67 active tuberculosis cases in Nunavut suggests a human—bear transmission route is unlikely to explain the high levels of MTBC that we observed^37^. These data perhaps indicate an unknown environmental reservoir of MTBC in Arctic ecosystems. Further work is needed to determine the species present in polar bears in our study, and to assess tissues for live bacteria.

We found low prevalence (18%) of *T. gondii*, with positive individuals widespread and no significant relationships evident for any biological or geographical factors (Fig. 1E). Given that *T. gondii* oocysts are shed solely by definitive felid hosts, lack of a relationship with human settlements implies that domestic animals are not the source of infection. The only known wild felid in the North American Arctic ecosystem is the Canadian lynx (*Lynx canadensis*), which only has modest range overlap with the polar bear, but dispersal of oocysts through snow-melt runoff and accumulation and transport in marine environments is well established^68^. Polar bears may be exposed to *T. gondii* through consumption of marine mammals such as seals, which have a relatively high prevalence (26–63%)^69^ in the eastern Canadian Arctic. While we detected a relatively low prevalence of *T. gondii* (18%) DNA, previous estimates of seroprevalence in polar bears (69.6%)^32^ show widespread exposure. It is common that serological estimates, which measure lifetime exposure, are higher than direct detection methods for microscopic cysts of *T. gondii* in small tissue samples^70^. As well, we did not survey tissues such as brain, a high predilection site for *T. gondii*^71^. As such, our use of skeletal muscle may underestimate *T. gondii* prevalence in the polar bear population, though as the most frequently consumed part of the animal, our findings present a more relevant portrayal of the associated human health risk.

We found 75% of individuals to be positive for *Trichinella* spp. (n = 51), with the highest prevalence in the south-central Arctic (94%; Seasonal ice ecoregion; Fig. 1F), and identified associations with sex, sexual maturity, and increasing waste accumulation (Supplementary material). We believe that tissue type contributed to the large differences in prevalence observed between the western (25—33%) and central Arctic (86—94%), with the low availability of muscle tissue (n = 4; the preferred predilection site for *Trichinella* spp.) in the western Arctic likely resulting in an underestimate of prevalence. As anticipated, adult bears were more likely to be positive, as risk of exposure to *Trichinella* spp. increases with every meal consumed, and infections are long-lived^41,72^. Similar observations are evident in the wolverine, another high trophic level predator and scavenger in the Canadian North^73^. The overall high prevalence of *Trichinella* spp. in polar bear populations and the relatively low prevalence in prey species have led to speculation on the role of cannibalism in maintaining rates of *Trichinella* spp.^41,74–78^. However, recent modeling suggests that polar bear consumption rates of marine mammals can account for the observed rates of *Trichinella* spp. infection and that other transmission pathways such as cannibalism play only a small role in prevalence^72^. Further, the observed association with human settlements identified here and by Pilfold et al., (2021) support that the scavenging of terrestrial waste (e.g., carcasses of human-hunted or trapped wildlife) may be a more common route of exposure for bears then presently understood^32^.

There were several associations observed between the assessed pathogens, with only two consistent across datasets: *E. rhusiopathiae*—*F. tularensis* and MTBC—*F. tularensis*. This suggests either that infection with one pathogen may create opportunity for infection with the other, or that there is another common predisposing factor for both pathogens that we did not include in our analysis. This may be particularly relevant for the relationship between MTBC and *F. tularensis*, as they demonstrated similar trends, (i.e., seasonality, waste accumulation, and geographic distribution). Conversely, these relationships could exist simply because *F. tularensis* is 21 times more likely to be present if an individual is infected with MTBC, and thus, some of the significant associations seen for *F. tularensis* may be a by-product of its association with MTBC. Without accompanying pathological and histological data these relationships are difficult to interpret.

While we recognize the limitations of our sampling in terms of statistical power, important patterns emerged from our analyses that merit further exploration. The univariate analyses highlighted statistically significant relationships between pathogens and predictors, while the recursive partitioning of variables allowed us to account for and identify potential relationships between predictor variables as well as the point of partitioning.

In general, hunters (specifically sport hunters) will preferentially target larger, healthier adult animals creating a potential bias in the available samples. Our dataset also includes non-hunted bears, animals euthanized and killed in defense of life and property, somewhat alleviating this bias. As well, the clinical status of the animals included in the analyses is unknown. Finally, detection of DNA of pathogens in tissues, especially the bacteria which are not thought to establish chronic tissue infections, should not be interpreted as active infection with viable pathogens. This limits our ability to determine the significance of these detections for both wildlife and human health.

Here we demonstrate that harvest-based monitoring of a sentinel species is a powerful tool in wildlife disease surveillance. Our work highlights and offers a solution for the need for a method to better characterize pathogen distributions and diversity in the Arctic. We generated baseline data on the geographic distributions and prevalence of the five zoonotic focal pathogens in the polar bear population and assessed the relevance of key demographic, biotic and abiotic factors as predictors for occurrence. Targeting several specific pathogens with highly sensitive digital droplet PCR assays allowed us to make observations about the pathogen community that may have otherwise been overlooked through broader mechanisms like 16S rRNA metabarcoding. Aside from highlighting the value of polar bears as sentinel species, our study also emphasizes their potential role in the distribution of pathogens within and between marine and terrestrial ecosystems. Our work lays the foundation for future investigations. We clearly must identify which species of MTBC is present, with work including microbial, genomic, pathological, and histological evaluations of animals near the areas of high prevalence. Evaluation of archived tissue samples using our ddPCR assay may tell us about the historical presence of these pathogens, including the role polar bears may (or may not) have played in the *E. rhusiopathiae* outbreak in the muskox population^19^. Further work is needed to identify the *F. tularensis* biovar present, *Trichinella* species, and *T. gondii* strains. Increased testing for bacterial pathogens in possible reservoir populations, especially rodents, would improve our understanding of the persistence and full life cycles of these pathogens in Arctic ecosystems.

Our research highlights the importance of community involvement and how support from northern governments can enable real-time monitoring of changes in the geographic distribution and prevalence of the focal pathogens. Expansion of our novel molecular tools to include additional pathogens can be done at the behest of communities or wildlife managers (*Pasteurella* multocida^79^; *Mycobacterium avium* subsp. *paratuberculosis*^80^, and *Brucella abortus*^33^). The implications of our assays extend beyond the polar bears and the Canadian Arctic, as the future validation of these methods on polar bear feces and the continued community-based collection of field samples would allow us an unprecedented opportunity to provide critical, real-time, and non-invasive, disease surveillance across the Arctic and beyond.

## Methods

### Sample collection

Harvest tissue sets containing liver, fat, skeletal muscle, and the lower intestine (with contents) were collected from subsistence harvested (n = 30), sport hunted (n = 9), killed in defense of life and property (DLP; n = 8), or euthanized (n = 1) bears by Inuit hunters between 2015 and 2019. Hunters were provided with sample kits containing sampling instructions, anatomical diagrams, bags, and labeling materials. Geographical coordinates for each sample were recorded and samples were frozen and sent first to territorial government agencies and then to Queen’s University, where they were preserved at − 20 °C (Level 2 facility at Queen’s University, Kingston, ON, Canada). Hunters were remunerated for their effort.

Our final sample set consisted of harvest tissue sets (n = 68 individual bears) containing liver (n = 64) and muscle (n = 50) tissue, for a total of 114 samples. Of the individual bears, 20 originated from the Northwest Territories and the remaining 48 from Nunavut (Fig. 1A). Samples were assigned to one of the ice ecoregions based on the location that the bear was harvested. These tissue sets were derived from the following ice ecoregions: the Convergent ice region (n = 15; western Arctic), the Divergent ice region (n = 4; western Arctic), the Archipelago ice region (n = 14; central Arctic), and the Seasonal ice region (n = 35; south-central Arctic; as defined by Amstrup et al., 2007)^81^. Not all tissue sets were complete and thus sample distribution by ice ecoregion is summarized in Table 1.

### Tissue preparation and detection of pathogen DNA using digital droplet PCR

The methods for the tissue preparation, assay design and execution are detailed in Tschritter et al.^9^. In brief, a multiplexed magnetic-capture step was used to concentrate target DNA from large quantities of host-tissue with biotinylated capture-oligonucleotides specific to our target sequences. This was followed by the molecular detection of target pathogen DNA using two multiplexed ddPCR assays. The first assay was triplexed to target the three bacterial targets (*E. rhusiopathiae*, *F. tularensis* and MTBC) within a single probe-based reaction. The second assay was duplexed to target the two muscle-encysting parasites (*T. gondii* and *Trichinella* spp.) within a single probe-based reaction. A positive (g-blocks™) and a negative control (nuclease-free water) were included in triplicate on each plate, in addition to the sequence-specific magnetic capture positive control. All primer and probe sequences are available in the supplementary material. Each sample and control were assessed in triplicate and the results analyzed using a QX200 Droplet Reader and the Quanta-Soft ™ Version 2.0.0 (Bio-Rad) software. A reaction was considered positive if two of three replicates had three times the number of positive droplets relative to the negative control.

### Statistical analyses of digital droplet PCR detection data

We assigned a detection status (presence or absence) for each focal pathogen based on the results of our ddPCR assays for muscle and liver samples independently; an individual was considered positive if a pathogen was detected in either its liver or muscle and negative if it was detected in neither. As detection rates vary across tissue types for different pathogens^10^ and not all individuals were represented by both tissues, all univariate analyses were done on three datasets: 1. individual bears (n = 68); 2. liver (n = 64); and 3. muscle (n = 50).

### Prevalence estimates by ice ecoregion

Pathogen prevalence estimates across ice ecoregions, and for each tissue set, were calculated using the function *epi.conf* in the R package ‘*epiR’*^82^. The standard error (std. error) of the mean difference and the confidence interval (95% CI) were calculated based on the Wilson Score Interval, recommended by Brown et al., (2001) for small sample sizes^83^.

### Univariate analyses

We first did a series of univariate tests to evaluate individual predictors of pathogen presence independently. We tested for associations between pathogen presence and proximity to nearest human settlement and waste accumulation in nearest settlement (m^3^; Table 2) using logistic regression analyses. We used the R package ‘*geosphere*’ version 1.5–18^84^ to estimate the distances between each bear harvest site and the 143 Arctic settlements^85^; we then selected the shortest distance— ‘distance to nearest settlement (km)’ as one of our predictors. We used publicly sourced data on waste disposal infrastructure across the Canadian Arctic^86^ to provide estimates of waste accumulation (m^3^) for each community landfill. These data (waste accumulation; m^3^) were associated with each bear based on the nearest settlement to the bear harvest site. To reduce the impact of a handful of high values for larger settlements, we standardized for waste accumulation by subtracting the mean for all settlements from each value and dividing by the standard deviation.

We used Fisher’s exact tests to test for differences in pathogen prevalence between bear age classes (subadult or adult), bear sex (male or female), and harvest seasons (summer or winter) for each of three datasets. All predictor variables are summarized and described in Table 2.

### Recursive partitioning of variables

We related the positive detection at the individual level (1 = presence/0 = absence) of *E. rhusiopathiae*, *F. tularensis*, MTBC, *T. gondii* and *Trichinella* spp. to predictors through the generation of decision trees (the recursive partitioning of predictor variables). Decision trees are non-parametric in nature and therefore don’t require the data to meet assumptions of normality and are able to account for and identify interactions between many predictor variables even with a small number of observations^87^. We tested for multicollinearity between model variables using the variance inflation factor (VIF), which measures the strength of correlation between predictor variables within a regression model and excluded factors that were highly correlated (VIF > 5). This meant excluding population size of the nearest settlement (VIF = 13.7; Pearson correlation coefficient = 0.97) in favour of retaining waste accumulation (m^3^) because we felt the latter metric better reflected the presence of transient residents (tourists, military personnel, transient workers etc.) and historical population trends that would otherwise not be represented in formal population census data. To assess the relative influence of interactions within and among biologic and geographic factors we first generated a generalized linear mixed-model (GLMM) tree (recursive partitioning based on binomial generalized linear mixed models) for each pathogen in the R package ‘*glmertree*’ (v 0.2–4)^88^. The biological predictors included were bear sex and bear age class (subadult vs adult; Table 2), and the geographic predictors included were harvest season (summer vs. winter), distance (km) to nearest human settlement, and waste accumulation in the nearest settlement (m^3^; Table 2). For the *glmertree* models we used the same constrained set of a priori models for each pathogen, balanced for equal representation of all variables (n = 63). We included ‘tissue type’, ‘harvest year’, and ‘ecoregion’ as random effects in each model, to account for the different tissue types (liver, muscle, or both) available for each bear, annual variation in pathogen presence, and differences due to sampling region. We then evaluated the inter-class correlation (ICC) of each of the random effect variables and compared the means of the clusters. If the ICC value of a variable was equal to zero and there was no difference between the means of the clusters, the variable was removed. For pathogens which had all the random effects removed, a conditional inference tree was generated using the *ctree* function in the R package ‘*partykit*’ (v 1.2–20)^89^, using the same set of predictor variables. The recursive partitioning method employed by *ctree* allows for imbalanced representation of predictors, allowing for the use of the full dataset (n = 68). Additional multivariate analyses are included in the Supplementary materials.

### Co-occurrence of pathogens

We use the R package ‘*CooccurrenceAffinity’*^90^ to estimate the probability of two pathogens co-occurring by chance (two-sided p-value for the equal-tailed test), across the three datasets (individual, muscle, and liver) along with more traditional (Jaccard’s index, Sørensen-Dice index, and the Simpson Index) and newer metrics (the affinity metric of co-occurrence)^91^. The p-values were adjusted using the sequential Bonferroni correction to account for multiple comparisons. The odds ratio and confidence interval (0.95; normal approximation) was calculated for the identified relationships using the ‘oddsratio’ function and small sample size adjustment in R package ‘*epitools’*^92^.

### Supplementary Information

Supplementary Information 1.

Supplementary Information 2.

Supplementary Information 3.

Supplementary Information 4.

Supplementary Information 5.

## Data Availability

Data are publicly accessible in our GitHub repository (10.5281/zenodo.8336170).
