# Supplementary material for: The geographic distribution, and the biotic and abiotic predictors of select zoonotic pathogen detections in Canadian polar bears
Source: Sci Rep. 2024 May 26;14:12027. doi: 10.1038/s41598-024-62800-x (PMC11128453; doi:10.1038/s41598-024-62800-x)
Supplement: Supplementary file 1 — Supplementary Information 1. [file 41598_2024_62800_MOESM1_ESM.pdf]

Supplementary materials for "Geographic distribution, and the biotic and abiotic predictors of select zoonotic pathogen detections in Canadian polar bears"

Supplementary T1. A summary of the genes targeted, and the primer and probe sequences used to identify each of five pathogens assessed in this manuscript.

Length	Type	Name	Primer/ Probe Sequence	Citation
Erysipelothrix rhusiopathiae (non-coding region)				
165 bp	Forward primer	ER_F[1]	ACCCTCTAATCGATATGCATCA	[1] (Pal et al., 2010)
	Reverse primer	ER_R[1]	ATTTCTCTAGCAGGTGATTTGG	
	Forward capture oligonucleotide	ER_(B)F	CGGATGATACACCCTCTAATCGATATGCATCA/BIOTIN-TEG/	
	Reverse capture oligonucleotide	ER_(B)R	AGGTTGCCTCATTTCTCTAGCAGGTGATTTGG/BIOTIN-TEG/	
	Probe	ER_PR[1]	/6-FAM/5-HEX/ TGTTGGACTACTAATCGTTTCGTT /BHQ1/	
Francisella tularensis (*FT Tul4* lipoprotein gene)				
212 bp	Forward primer	FT_F [2]	TGGCGAGTGATACTGCTTG	[2] (Junhui et al., 1996)
	Reverse primer	FT_R [2]	CGCTAAACCTGCGATTGAT	[2] (Lamps et al., 2004)
	Forward capture oligonucleotide	FT_(B)F	GTTGATTTTATGGCGAGTGATACTGCTTG/BIOTIN-TEG/	
	Reverse capture oligonucleotide	FT_(B)R	TAGAACAGCTCGCTAAACCTGCGATTGAT/BIOTIN-TEG/	
	Probe	FT_PR	/6-FAM/AATCGTAATGTTAGCTGTATCATCATT/MGB-NFQ/	
MTBC, *Mycobacterium tuberculosis* complex (*mpb70* gene)				
133 bp	Forward primer	MTBC_F [3]	CTCAATCCGCAAGTAAACC	[3] (Lorente-Leal et al., 2019)
	Reverse primer	MTBC_R [3]	TCAGCAGTGACGAATTGG	
	Forward capture oligonucleotide	MTBC_(B)F	GTCGGGCCAGCTCAATCCGCAAGTAAACC/BIOTIN-TEG/	
	Reverse capture oligonucleotide	MTBC_(B)R	CAGGATGCTGGTCAGCAGTGACGAATTGG/BIOTIN-TEG/	
	Probe	MTBC_PR [3]	/5-HEX/CTCAACAGCGGTCAGTACACGGT/BHQ1/	

Toxoplasma gondii (DNA repeat element)

164 bp	Forward primer	TOX_F [4]	AGGAGAGATATCAGGACTGTAG	[4] (Reischl et al., 2003)
	Reverse primer	TOX_R [4]	GCGTCGTCTCGTCTGGATCG	[5] (Opsteegh et al., 2010)
	Forward capture oligonucleotide	TOX_(B)F [5]	/BIOTIN-TEG/CTTGGAGCCACAGAAGGGACAGAAGTCGAAGGGGACTACAGACGCGATGCCGCTCCTCCAGCCGTCTTGG	
	Reverse capture oligonucleotide	TOX_(B)R [5]	/BIOTIN-TEG/AAGCCTCCGACTCTGTCTCCCTCGCCCTCTTCTCCACTCTTCAATTCTCTCCGCCATCACCACGAGGAA	
	Probe	TOX_PR [5]	/6-FAM+ZEN/CCGGCTTGGCTGCTTTTCCT/IBFQ/	

Trichinella spp. (mitochondrial *rrnS* gene)

195 bp	Forward primer	TR_F [6]	GGTCCTCCTTCCAGAAGATCTACTTTG	[6] (Cuttell et al., 2012)
	Reverse primer	TR_R [6]	CATGGTTAGGTGAGATATTGCCTGC	
	Forward capture oligonucleotide	TR_(B)F	CTCTAGGCTAGGTCCTCCTTCCAGAAGATCTACTTTG/BIOTIN-TEG/	
	Reverse capture oligonucleotide	TR_(B)R	AGATATAAATCATGGTTAGGTGAGATATTGCCTGC/BIOTIN-TEG/	
	Probe	TR_PR	/5-HEX/AGGGTGACGGGCAATATGTGCATA/BHQ1/	

* BIOTIN-TEG capture-oligonucleotides labelled with a biotin-triethylene-glycol spacer arm
*6-FAM fluorescent reporter dye
*5-HEX fluorescent reporter dye
*BHQ-1 Black-hole quencher; non-fluorescent chromophore
*IBFQ Iowa black FQ
*MGB-NFQ Minor groove binding – Non-fluorescent quencher modification; used to increase T_m

Supplementary T2. A summary of the molecular results for each tissue set, with (-) indicating no detection of the target pathogen, (+) indicating a positive detection for the target pathogen, and NA indicating tissue unavailability for the test.

	E. rhusiopathiae		*F. tularensis*		MTBC		*T. gondii*		*Trichinella* spp.	
	Liver	Muscle	Liver	Muscle	Liver	Muscle	Liver	Muscle	Liver	Muscle
1	-	NA	-	NA	-	NA	-	-	+	NA
2	-	NA	-	NA	-	NA	-	-	+	NA
3	-	NA	-	NA	+	NA	-	-	+	NA
4	-	NA	-	NA	-	NA	-	-	-	NA
5	-	NA	-	NA	-	NA	-	-	-	NA
6	-	-	-	-	-	+	-	-	-	-
7	-	-	-	-	-	-	-	-	+	+
8	-	NA	-	NA	-	NA	-	-	+	NA
9	-	NA	-	NA	-	NA	-	-	-	NA
10	-	NA	-	NA	-	NA	-	-	-	NA
11	-	NA	-	NA	-	NA	-	-	-	NA
12	-	NA	-	NA	-	NA	-	-	-	NA
13	-	NA	-	NA	-	NA	-	-	-	NA
14	-	NA	-	NA	-	NA	-	-	-	NA
15	-	NA	-	NA	-	NA	-	-	+	NA
16	-	NA	-	NA	-	NA	-	-	-	NA
17	+	NA	-	NA	-	NA	-	-	-	NA
18	+	+	-	-	-	-	-	+	-	+
19	+	+	-	-	-	-	-	+	-	-
20	+	NA	-	NA	-	NA	-	NA	-	NA
21	-	-	+	-	-	-	-	-	-	-
22	-	+	-	-	-	-	-	+	-	+
23	-	+	-	-	-	-	-	-	-	-
24	+	+	-	-	-	-	-	+	-	+
25	-	-	-	-	-	-	-	-	-	+
26	+	+	-	-	-	-	-	-	+	+
27	+	-	-	-	-	+	-	-	+	+
28	+	-	-	-	-	-	-	-	+	+
29	-	-	-	-	-	+	-	-	-	-
30	-	-	-	-	-	+	-	-	+	+
31	-	-	-	-	-	-	-	-	+	+
32	-	NA	-	NA	-	NA	-	NA	+	NA
33	-	-	-	-	-	-	-	-	-	-
34	-	-	-	-	-	-	-	-	-	+
35	NA	-	NA	-	NA	-	NA	-	NA	+

36	-	-	-	-	-	-	-	-	-	+
37	+	-	-	-	-	-	-	+	-	+
38	NA	-	NA	-	NA	-	NA	-	NA	+
39	-	NA	-	NA	-	NA	-	NA	+	NA
40	-	-	-	-	-	-	-	-	+	+
41	-	-	-	-	-	-	-	-	+	+
42	-	-	-	-	-	-	-	-	-	+
43	-	-	-	-	-	-	-	-	-	+
44	-	-	-	-	-	-	-	-	-	+
45	-	-	-	-	-	-	-	-	-	+
46	-	-	-	-	-	-	-	-	-	+
47	-	-	-	-	-	-	-	-	-	+
48	NA	-	NA	-	NA	-	NA	-	NA	+
49	-	-	-	-	-	-	-	+	+	+
50	-	-	-	-	-	+	-	-	+	+
51	-	-	-	-	-	-	-	-	-	+
52	-	-	-	-	-	-	-	-	+	+
53	-	-	-	-	-	+	-	-	+	+
54	NA	+	NA	+	NA	+	NA	-	NA	+
55	+	-	-	-	+	+	-	-	+	+
56	-	+	-	+	+	+	-	-	+	+
57	-	+	-	-	+	+	-	+	+	+
58	-	+	-	-	+	+	-	-	-	+
59	-	+	-	-	+	+	-	-	+	+
60	-	+	-	+	+	+	-	-	+	+
61	-	+	-	+	+	+	-	-	+	+
62	-	-	-	+	+	+	-	-	+	+
63	-	+	-	-	+	+	+	-	+	+
64	+	+	+	+	+	+	-	+	+	+
65	+	+	+	+	+	+	-	-	+	+
66	+	+	+	+	+	+	-	+	+	+
67	+	+	+	+	+	+	-	+	+	+
68	+	+	+	+	+	+	-	+	+	+

Multivariate analyses methods and results

We related the positive detection at the individual level (1=presence/0=absence) of *E. rhusiopathiae*, *F. tularensis*, MTBC, *T. gondii* and *Trichinella* spp. to predictors using binomial (logit link) generalized linear mixed models in the R package '*lme4'* (v 1.1-31; Bates et al., 2015). We used the same constrained set of a priori models for each pathogen, balanced for equal representation of all variables. We tested for multicollinearity between model variables using the variance inflation factor (VIF), which measures the strength of correlation between predictor variables within a regression model and excluded factors that were highly correlated (VIF >5). This meant excluding population size of the nearest settlement (VIF=13.7; Pearson correlation coefficient =0.97) in favour of retaining waste accumulation (m^3), because we felt the latter metric better reflected the presence of transient residents (tourists, military personnel, transient workers etc.) and historical population trends that would otherwise not be represented in formal population census data. We included 'tissue type' and 'harvest year' as a random effect in each model, to account for the different tissue types (liver, muscle, or both) available for each bear, and annual variation in pathogen presence. To assess the relative influence of interactions within and among biologic and geographic factors we used Akaike information criteria corrected for small sample sizes (AIC_c). Biological and geographic predictors were first assessed independently to identify top predicting factors, which we defined as any factor with an AIC_c w_t ≥ the AIC_c w_t of the random effect model. The biological predictors included bear sex and bear age class (subadult vs adult; Table 4.2). Geographic predictors included harvest season (summer vs. winter), distance (km) to nearest human settlement, and waste accumulation in the nearest settlement (m^3; Table 4.2). Top factors were then evaluated independently and in all plausible combinations of additive and interaction effects within the groups and the models compared. The top model(s) was selected based on the lowest AIC_c value and a delta AIC_c<2.

The results of the multivariate analyses are presented in Supplementary T3. Waste accumulation (m^3) in the nearest settlement was a top predictor for *E. rhusiopathiae* (AIC_c w_t =0.56) and harvest season (summer; AIC_c w_t =0.85) a top predictor for *F. tularensis*. Both harvest season (summer; AIC_c w =0.96) and proximity to nearest settlement (km; AIC_c w_t =0.72) were top factors in the detection of the MTBC. The top multivariate model for MTBC was an interaction between harvest season and distance to nearest settlement (km), which had an AIC_c w_t =0.82. Identifying that when controlling for season, tissue type, and harvest year the proximity to human settlement is an important predictor for MTBC detection. One of the MTBC models ('Model 3: Harvest season + Distance to nearest settlement (km)') failed to converge. Bear age class (adults; AIC_c w_t =0.56) was a top predictor for the occurrence of *Trichinella* spp. No factors for *T. gondii* had AIC_c w_t ≥ the AIC_c w_t of the random effect model.

Supplementary T3. A summary of the resulting multivariate models (controlling for random effect of 'tissue type' and 'year'), the number of estimated parameters (K), Akaike information criterion corrected for small sample size (AIC_c), the difference in AIC_c value relative to the best model (Delta AIC_c), the relative likelihood of the model (ModelLik), the Akaike weight (AIC_c W_t), the log likelihood (LL), and the cumulative model Akaike weights (Cum. W_t). The MTBC 'Model 3: Harvest season + Distance to nearest settlement' failed to converge.

Top Models	K	AICc	Delta AICc	ModelLik	AICc Wt	LL	Cum. Wt
E. rhusiopathiae							
Model 2: Waste accumulation in the nearest settlement	4	82.26	0.00	1.00	0.56	-36.78	0.56
Model 1: Random effect of tissue type & year	3	82.73	0.47	0.79	0.44	-38.16	1.00
F. tularensis							
Model 2: Harvest season	4	57.71	0.00	1.00	0.85	-24.51	0.85
Model 1: Random effect of tissue type & year	3	61.25	3.54	0.17	0.15	-27.42	1.00
MTBC							
Model 4: Interaction Harvest season * Distance to nearest settlement	6	65.49	0.00	1.00	0.82	-25.99	0.82
Model 3: Harvest season + Distance to nearest settlement	5	69.84	4.35	0.11	0.09	-29.40	0.91
Model 1: Harvest season	4	70.19	4.70	0.10	0.08	-30.75	0.99
Model 2: Distance to nearest settlement	4	74.62	9.13	0.01	0.01	-32.97	1.00
Model 5: Random effect of tissue type & year	3	76.53	11.04	0.00	0.00	-35.06	1.00
T. gondii							
** No factors were considered top predictors*							
Trichinella spp.							
Model 2: Bear age	4	57.75	0.00	1.00	0.56	-24.53	0.56
Model 1: Random effect of tissue type & year	3	58.20	0.45	0.80	0.44	-25.89	1.00

Supplementary T4. Co-occurrences (by individual) of the positive molecular detections for each of the target pathogens, with an asterisk indicating a significant (p-value < 0.05) association between the two identified pathogens that was upheld independently across the three datasets (individual, muscle, and liver).

	E. rhusiopathiae	*F. tularensis*	MTBC	*T. gondii*	*Trichinella* spp.
E. rhusiopathiae	X	**13%***	22%	16%	31%
F. tularensis		X	**15%***	6%	15%
MTBC			X	9%	28%
T. gondii				X	16%

Supplementary T5. A summary of the pairwise co-occurrence analyses conducted for the three data sets (individual, muscle, and liver), including the observed and expected co-occurrence statistics, the p-value, the corrected p-value based on the Bonferroni correction for multiple comparisons, the 95% confidence interval, the affinity metric of co-occurrence (Alpha MLE; as described in Mainali & Slud, 2022), Jaccard's Index (*J*), Sørensen-Dice Index, and the Simpson Index.

	**A**	**B**	**A (*n*)**	**B (*n*)**	**Total (N)**	**Obs. Co-occurrence**	**Exp. Co-occurrence**	**P-value**	**Corr. P-value**	**CI (0.95)**	**Alpha MLE**	**Jaccard's Index**	**Sørensen-Dice Index**	**Simpson Index**
Individual														
**1**	**ER**	**FT**	**25**	**11**	**68**	**9**	**4.044**	**0.0013**	**0.013**	**[0.821, 4.363]**	**2.404**	**0.333**	**0.5**	**0.818**
**2**	**ER**	**MTBC**	**25**	**20**	**68**	**15**	**7.353**	**5.33E-05**	**5.33E-04**	**[1.083, 3.651]**	**2.387**	**0.5**	**0.667**	**0.75**
**3**	**ER**	**TOX**	**25**	**12**	**68**	**11**	**4.412**	**2.70E-05**	**2.70E-04**	**[1.516, 6.573]**	**3.439**	**0.423**	**0.595**	**0.917**
4	ER	TRICH	25	51	68	21	18.75	0.2514	1	[-0.436, 2.134]	0.811	0.382	0.553	0.84
**5**	**FT**	**MTBC**	**11**	**20**	**68**	**10**	**3.235**	**5.89E-06**	**5.89E-05**	**[1.784, 6.932]**	**3.772**	**0.476**	**0.645**	**0.909**
6	FT	TOX	11	12	68	4	1.941	0.0942	0.942	[-0.26, 2.79]	1.229	0.211	0.348	0.364
7	FT	TRICH	11	51	68	10	8.25	0.2679	1	[-0.619, 4.484]	1.346	0.192	0.323	0.909
8	MTBC	TOX	20	12	68	6	3.529	0.159	1	[-0.354, 2.556]	1.08	0.231	0.375	0.5
9	**MTBC**	**TRICH**	**20**	**51**	**68**	**19**	**15**	**0.0145**	0.145	**[0.24, 5.33]**	**2.227**	**0.365**	**0.535**	**0.95**
10	TOX	TRICH	12	51	68	11	9	0.1708	1	[-0.463, 4.595]	1.465	0.212	0.349	0.917
Muscle														
**1**	**ER**	**FT**	**20**	**10**	**50**	**9**	**4**	**5.00E-04**	**0.005**	**[1.034, 6.271]**	**3.098**	**0.429**	**0.6**	**0.9**
**2**	**ER**	**MTBC**	**20**	**19**	**50**	**13**	**7.6**	**0.0025**	**0.025**	**[0.662, 3.31]**	**1.957**	**0.5**	**0.667**	**0.684**
**3**	**ER**	**TOX**	**20**	**11**	**50**	**10**	**4.4**	**2.00E-04**	**0.002**	**[1.263, 6.462]**	**3.291**	**0.476**	**0.645**	**0.909**
4	ER	TRICH	20	44	50	18	17.6	1	1	[-1.469, 2.42]	0.319	0.391	0.562	0.9
**5**	**FT**	**MTBC**	**10**	**19**	**50**	**10**	**3.8**	**8.99E-06**	**8.99E-05**	**[1.938, 8.517]**	**8.517**	**0.526**	**0.69**	**1**
6	FT	TOX	10	11	50	4	2.2	0.1966	1	[-0.432, 2.647]	1.118	0.235	0.381	0.4
7	FT	TRICH	10	44	50	10	8.8	0.3271	1	[-1.175, 8.517]	8.517	0.227	0.37	1
8	MTBC	TOX	19	11	50	5	4.18	0.7271	1	[-0.979, 1.886]	0.389	0.2	0.333	0.455
9	MTBC	TRICH	19	44	50	18	16.72	0.3873	1	[-0.972, 4.442]	1.221	0.4	0.571	0.947
10	TOX	TRICH	11	44	50	10	9.68	1	1	[-1.92, 3.627]	0.379	0.222	0.364	0.909

Liver

**1**	**ER**	**FT**	**14**	**6**	**64**	**5**	**1.312**	**0.0014**	**0.014**	**[0.998, 6.47]**	**3.225**	**0.333**	**0.5**	**0.833**
2	ER	MTBC	14	15	64	6	3.281	0.0749	0.749	[-0.234, 2.559]	1.206	0.261	0.414	0.429
3	ER	TOX	14	1	64	0	0.219	1	1	[-9.011, 4.217]	-9.011	0	0	0
4	ER	TRICH	14	32	64	9	7	0.2469	1	[-0.57, 1.99]	0.736	0.243	0.391	0.643
**5**	**FT**	**MTBC**	**6**	**15**	**64**	**5**	**1.406**	**0.002**	**0.02**	**[0.895, 6.345]**	**3.107**	**0.312**	**0.476**	**0.833**
6	FT	TOX	6	1	64	0	0.094	1	1	[-9.011, 5.213]	-9.011	0	0	0
7	FT	TRICH	6	32	64	5	3	0.1961	1	[-0.41, 4.926]	1.724	0.152	0.263	0.833
8	MTBC	TOX	15	1	64	1	0.234	0.2344	1	[-1.761, 9.011]	9.011	0.067	0.125	1
**9**	**MTBC**	**TRICH**	**15**	**32**	**64**	**14**	**7.5**	**0.0002**	**0.002**	**[1.128, 6.25]**	**3.137**	**0.424**	**0.596**	**0.933**
10	TOX	TRICH	1	32	64	1	0.5	0.5	1	[-2.944, 9.011]	9.011	0.031	0.061	1

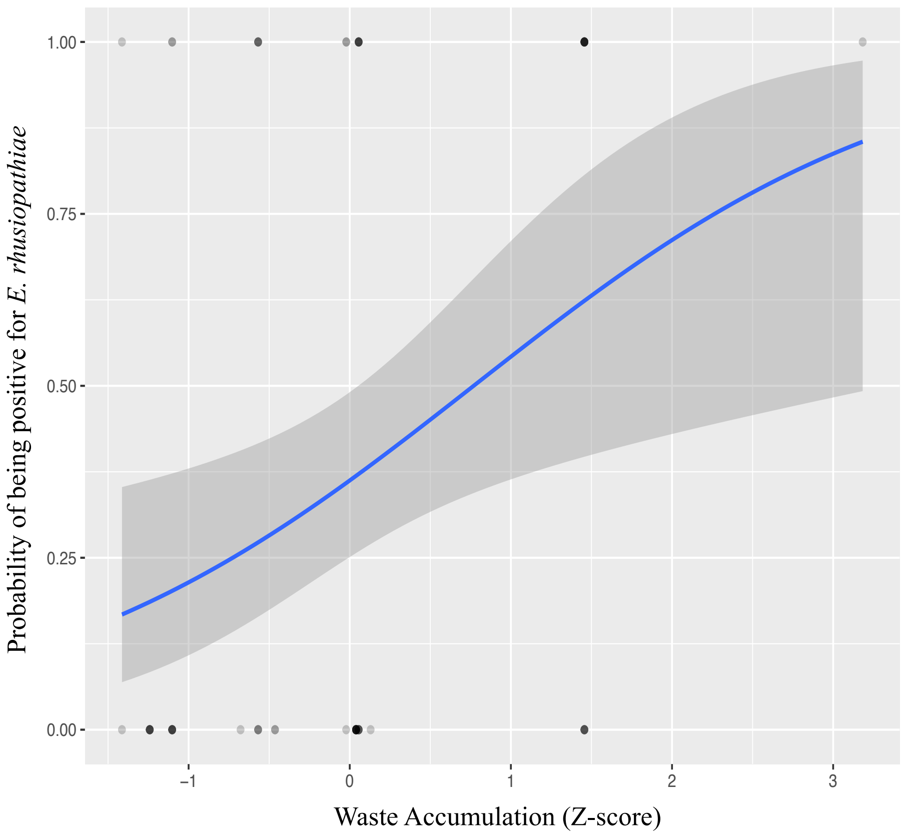

Supplementary F1. A logistic regression model displaying the probability of being positive for *E. rhusiopathiae* relative to the amount of waste accumulation in the nearest community (m^3; mean-centered).

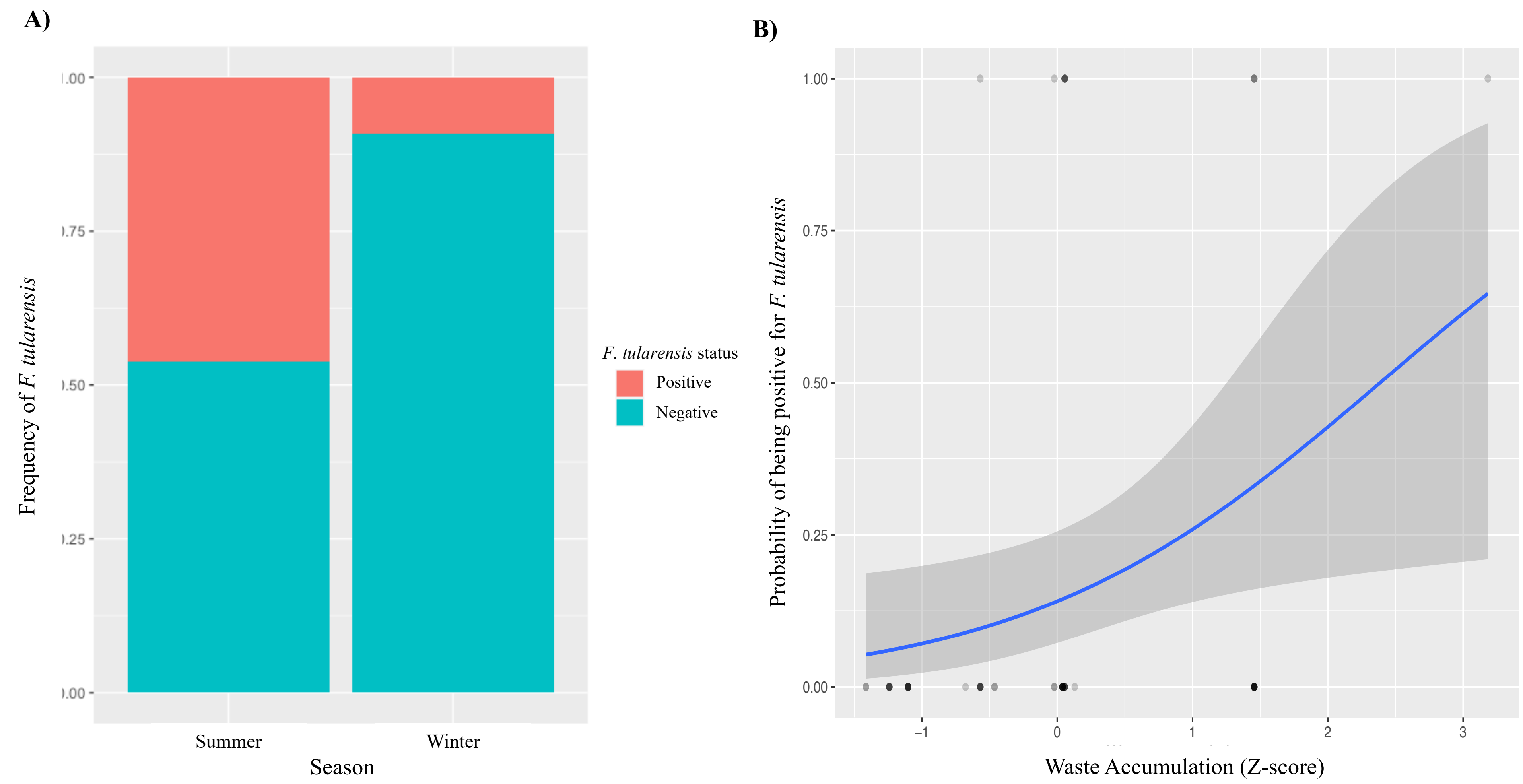

Supplementary F2. **(A)** A bar graph displaying the seasonal distribution (harvest season) of samples positive and negative for *F. tularensis.* As well as a logistic regression model **(B)** displaying the probability of being positive for *F. tularensis* relative to the amount of waste accumulation in the nearest community (m^3; mean-centered).

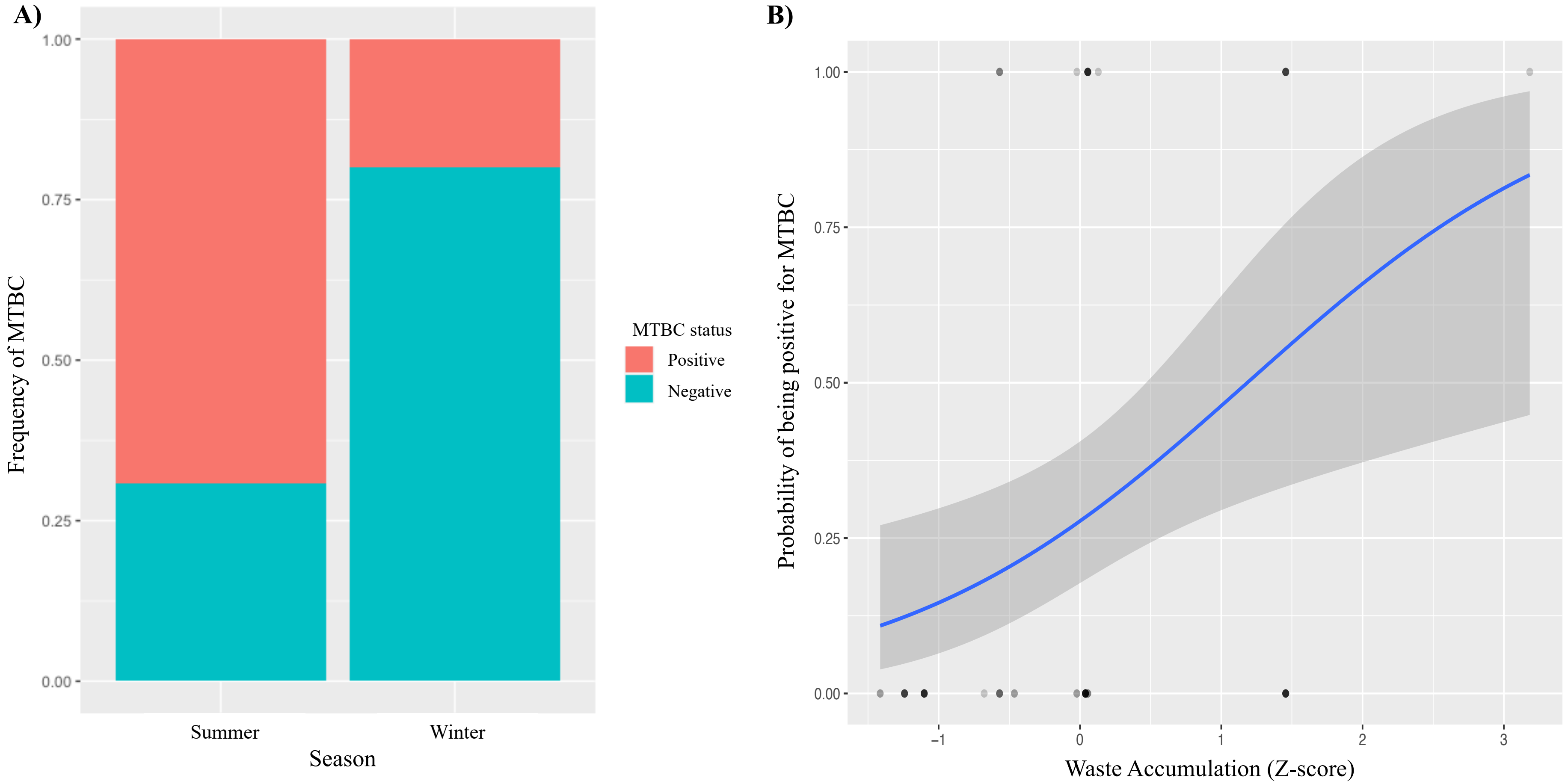

Supplementary F3. **(A)** A bar graph displaying the seasonal distribution (harvest season) of samples positive and negative for MTBC. As well as a logistic regression model **(B)** displaying the probability of being positive for MTBC relative to the amount of waste accumulation in the nearest community (m^3; mean-centered).

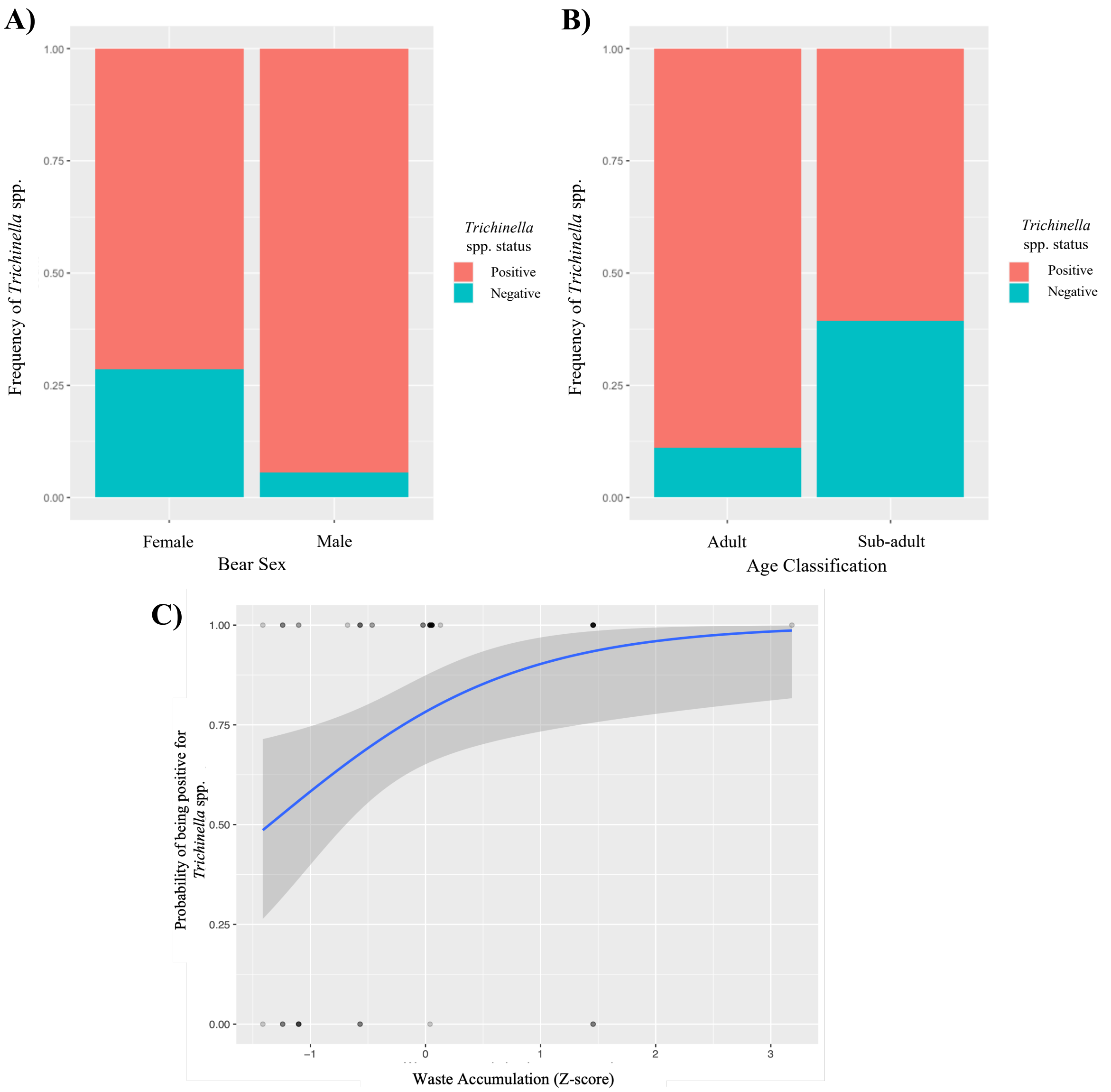

Supplementary F4. Two column graphs displaying **(A)** the sex distribution of the muscle tissue samples and **(B)** the age distribution of individual's positive and negative for *Trichinella* spp. As well as a logistic regression model displaying the probability of being positive for *Trichinella* spp. relative to **(C)** the amount of waste accumulation in the nearest community (m^3; mean-centered).

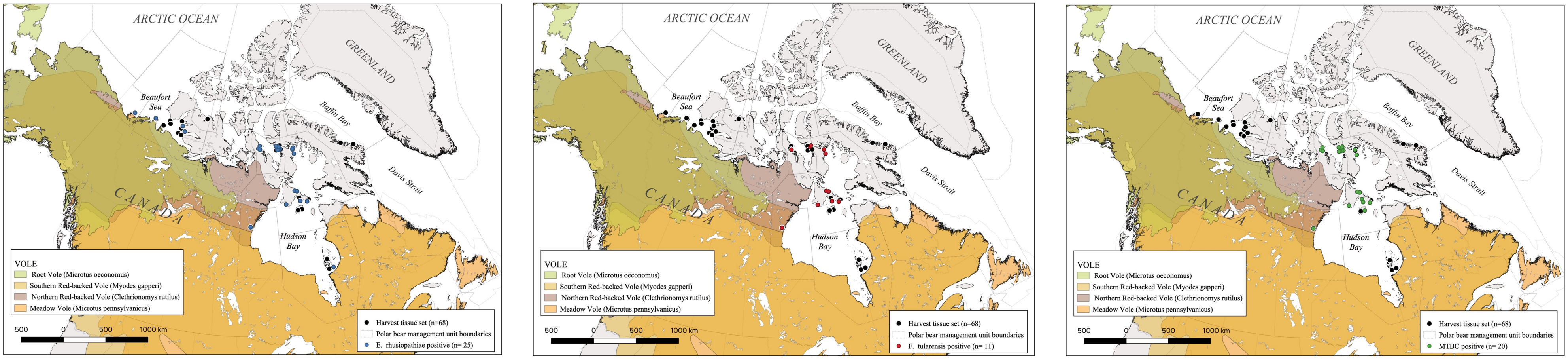

Supplementary F5. Three maps depicting the distributions of the positive and negative samples for each bacterial pathogen **(A)** *E. rhusiopathiae*, **(B)** *F. tularensis*, and **(C)** MTBC, and relative to the range distribution of the four species of vole known to occur in the Canadian Arctic (*M. oeconomus*, *M. gapperi*, *C. rutilus*, and *M. pennsylvanicus*). These maps were generated using free and open source QGIS software (*v*3.30.3; https://qgis.org).

References

Bates, D., Mächler, M., Bolker, B., & Walker, S. (2015). Fitting Linear Mixed-Effects Models Usinglme4. *Journal of Statistical Software*, *67*(1), 1-48. https://doi.org/10.18637/jss.v067.i01

Brown, L. D., Cai, T. T., & DasGupta, A. (2001). Interval estimation for a binomial proportion. *Statistical Science*, *16*(2), 101-117. https://doi.org/10.1214/ss/1009213286

Cuttell, L., Corley, S. W., Gray, C. P., Vanderlinde, P. B., Jackson, L. A., & Traub, R. J. (2012). Real-time PCR as a surveillance tool for the detection of Trichinella infection in muscle samples from wildlife. *Vet Parasitol*, *188*(3-4), 285-293. https://doi.org/10.1016/j.vetpar.2012.03.054

Junhui, Z., Ruifu, Y., Jianchun, L., Songle, Z., Meiling, C., Fengxiang, C., & Hong, C. (1996). Detection of Francisella tularensis by the polymerase chain reaction. *Journal of Medical Microbiology*, *45*(6), 477-482. https://doi.org/10.1099/00222615-45-6-477

Lamps, L. W., Havens, J. M., Sjostedt, A., Page, D. L., & Scott, M. A. (2004). Histologic and molecular diagnosis of tularemia: a potential bioterrorism agent endemic to North America. *Mod Pathol*, *17*(5), 489-495. https://doi.org/10.1038/modpathol.3800087

Lorente-Leal, V., Liandris, E., Castellanos, E., Bezos, J., Dominguez, L., de Juan, L., & Romero, B. (2019). Validation of a Real-Time PCR for the Detection of Mycobacterium tuberculosis Complex Members in Bovine Tissue Samples. *Front Vet Sci*, *6*, 1-9. https://doi.org/10.3389/fvets.2019.00061

Mainali, K. P., & Slud, E. (2022). CooccurrenceAffinity: An R package for computing a novel metric of affinity in co-occurrence data that corrects for pervasive errors in traditional indices. *Unpublished*, 1-34. https://doi.org/10.1101/2022.11.01.514801

Opsteegh, M., Langelaar, M., Sprong, H., den Hartog, L., De Craeye, S., Bokken, G., Ajzenberg, D., Kijlstra, A., & van der Giessen, J. (2010). Direct detection and genotyping of Toxoplasma gondii in meat samples using magnetic capture and PCR. *Int J Food Microbiol*, *139*(3), 193-201. https://doi.org/10.1016/j.ijfoodmicro.2010.02.027

Pal, N., Bender, J. S., & Opriessnig, T. (2010). Rapid detection and differentiation of Erysipelothrix spp. by a novel multiplex real-time PCR assay. *J Appl Microbiol*, *108*(3), 1083-1093. https://doi.org/10.1111/j.1365-2672.2009.04560.x

Reischl, U., Bretagne, S. p., Krüger, D., Ernault, P., & Costa, J.-M. (2003). Comparison of two DNA targets for the diagnosis of Toxoplasmosis by real-time PCR using fluorescence resonance energy transfer hybridization probes. *BMC Infectious Diseases*, *3*(7), 1-9. https://doi.org/10.1186/1471-2334-3-7
