# Supplementary figures and images for: The geographic distribution, and the biotic and abiotic predictors of select zoonotic pathogen detections in Canadian polar bears
Source: Sci Rep. 2024 May 26;14:12027. doi: 10.1038/s41598-024-62800-x (PMC11128453; doi:10.1038/s41598-024-62800-x)

Probability of being positive for *E. rhusiopathiae*

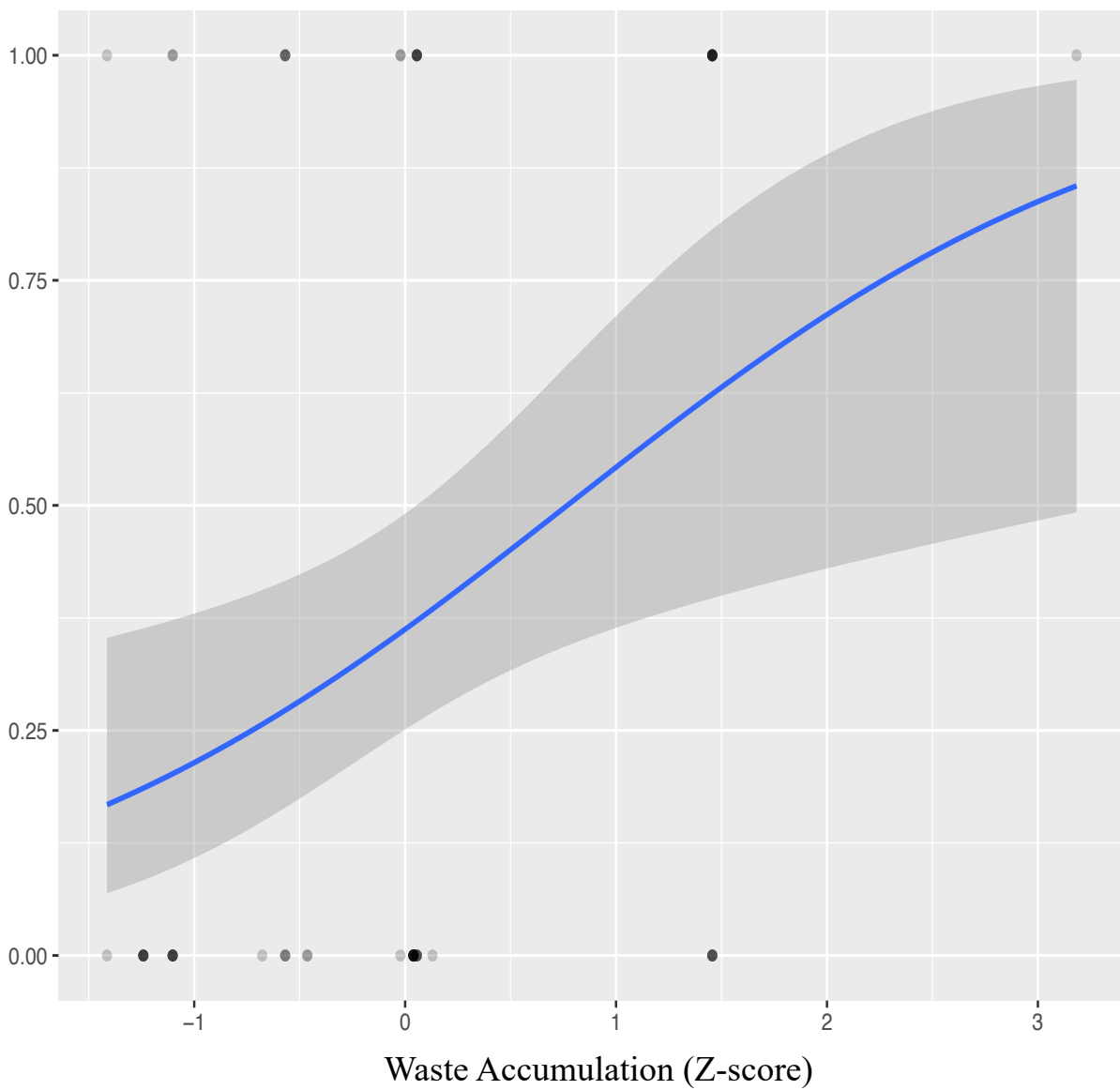

Supplement: Supplementary file 2 — Supplementary Information 2. [file 41598_2024_62800_MOESM2_ESM.pdf]

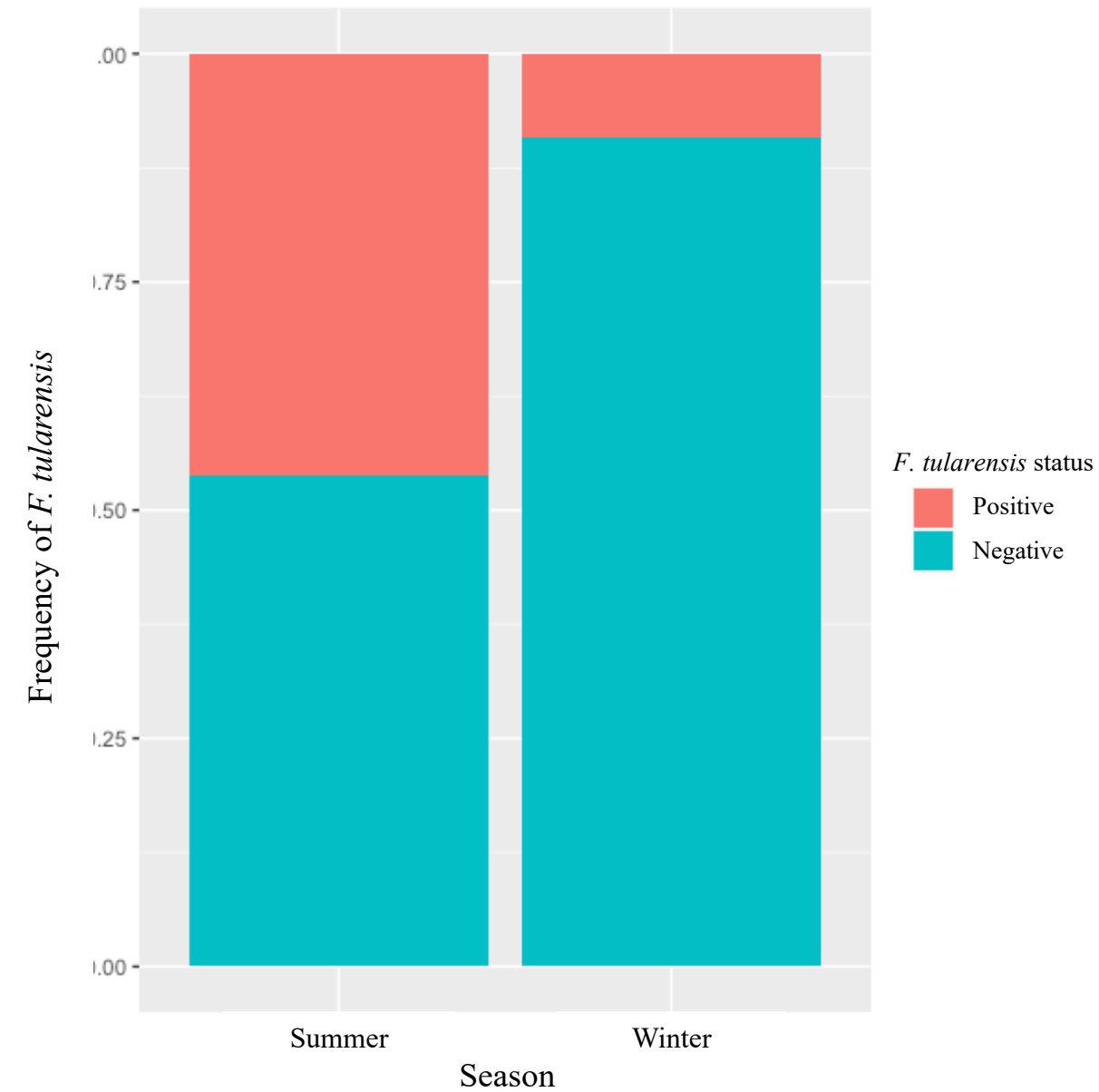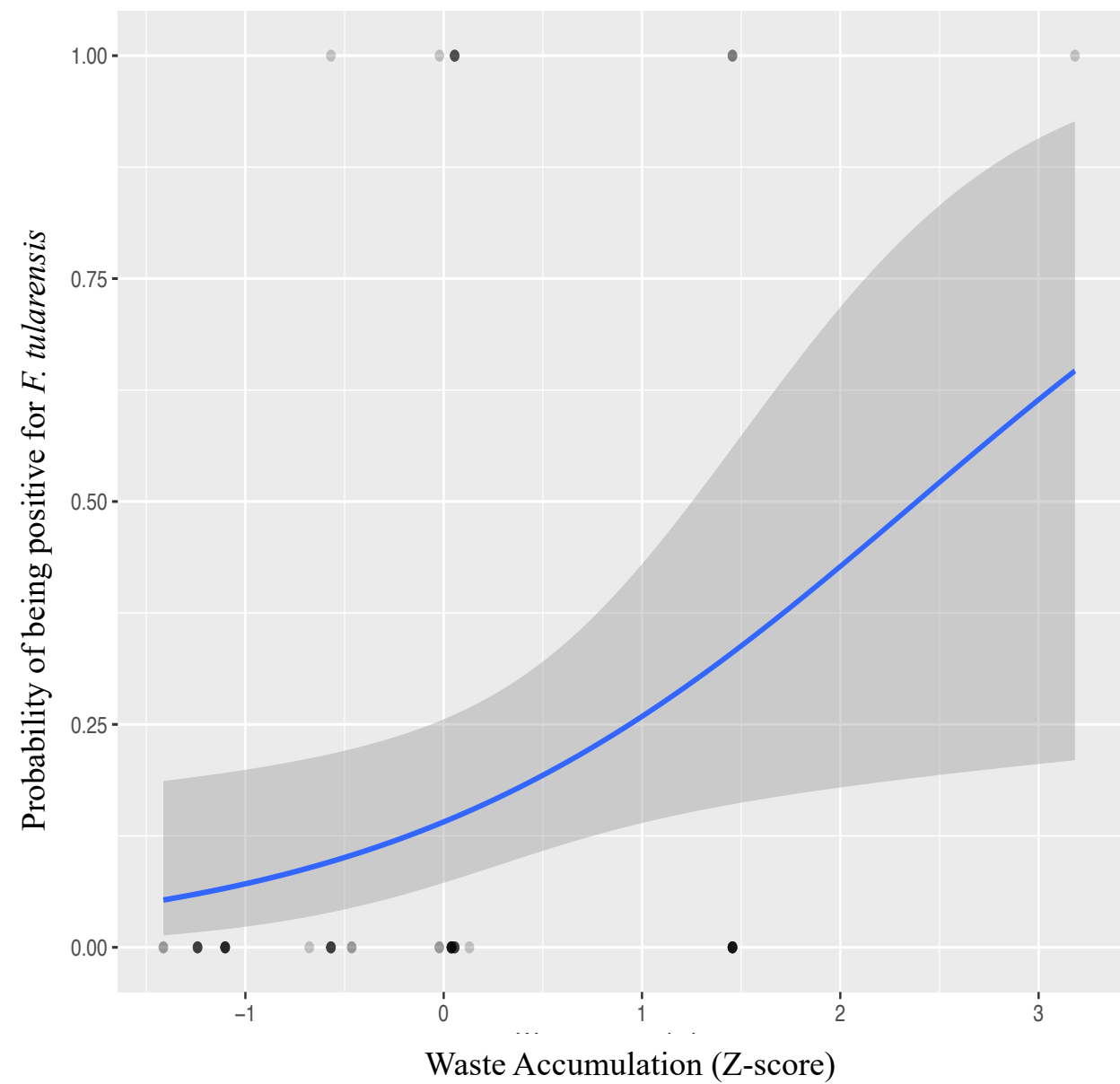

Supplement: Supplementary file 3 — Supplementary Information 3. [file 41598_2024_62800_MOESM3_ESM.pdf]

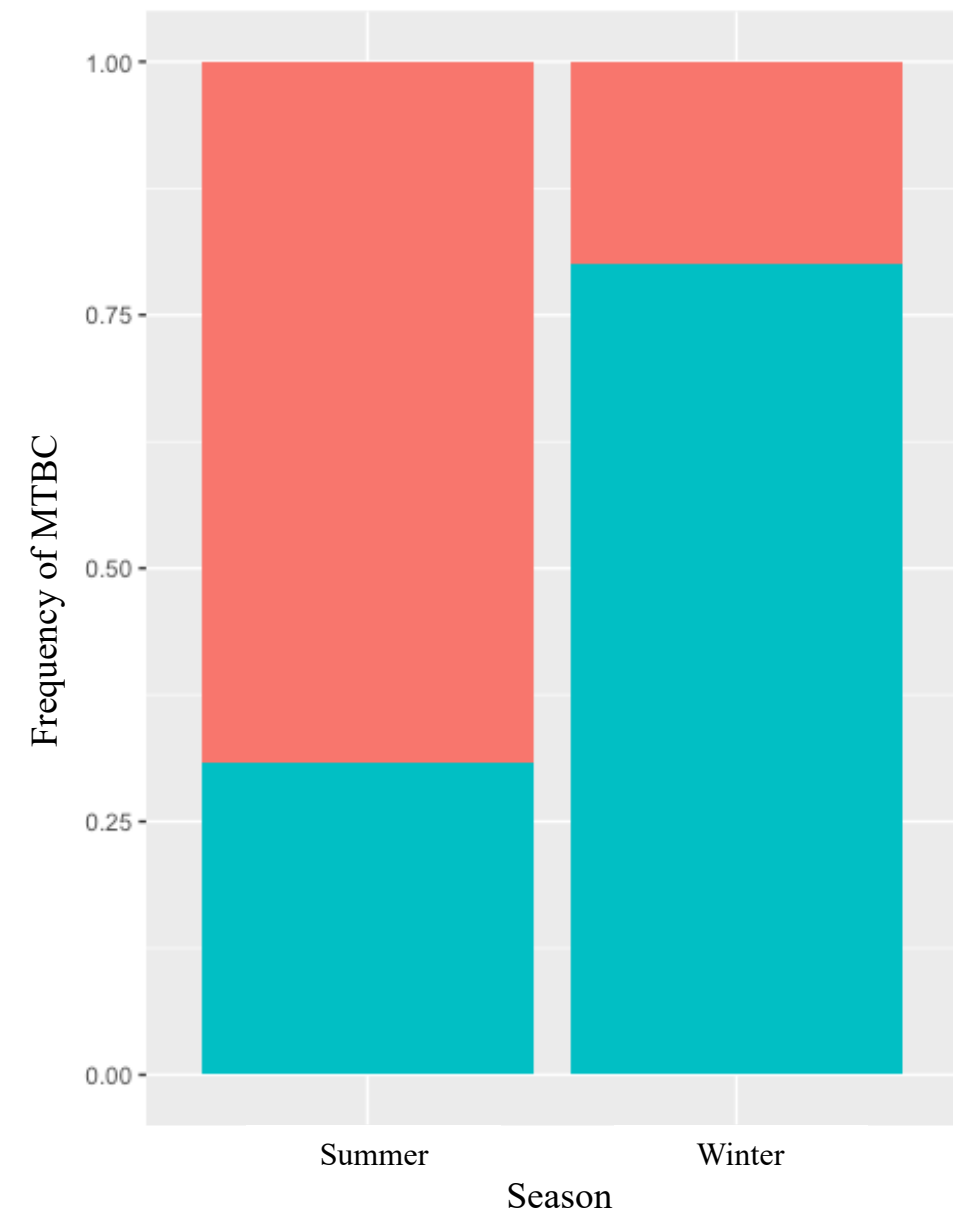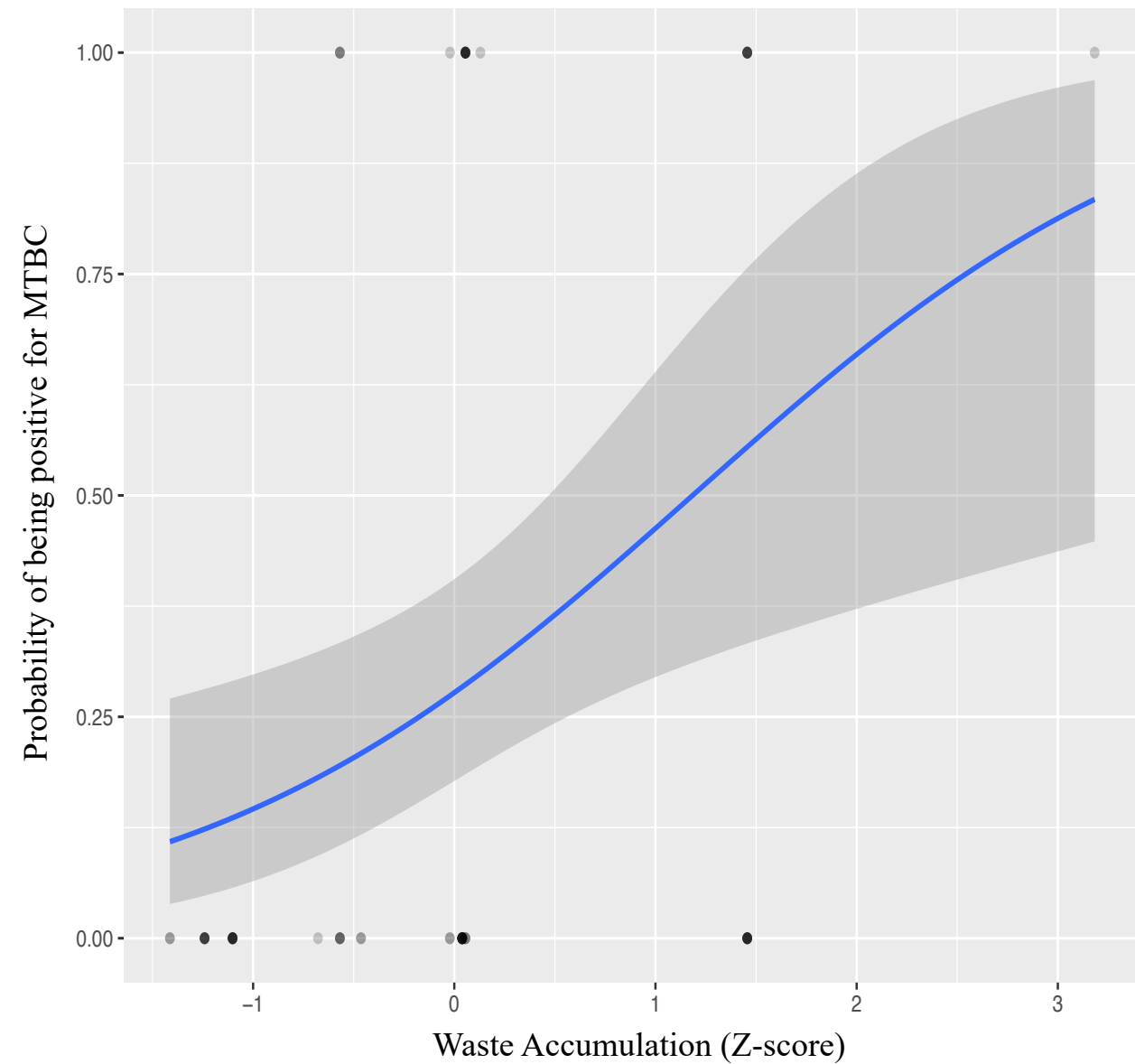

Supplement: Supplementary file 4 — Supplementary Information 4. [file 41598_2024_62800_MOESM4_ESM.pdf]

Frequency of *Trichinella* spp.

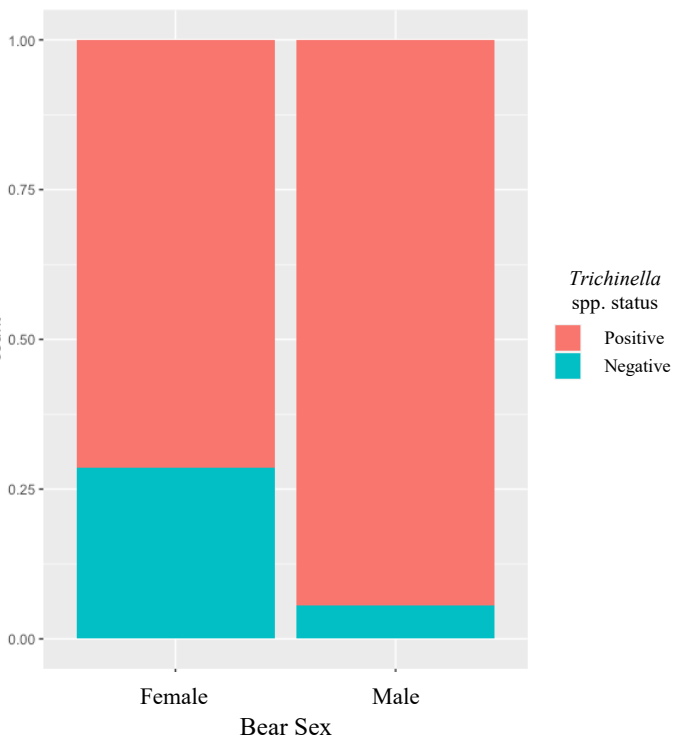

Frequency of *Trichinella* spp.

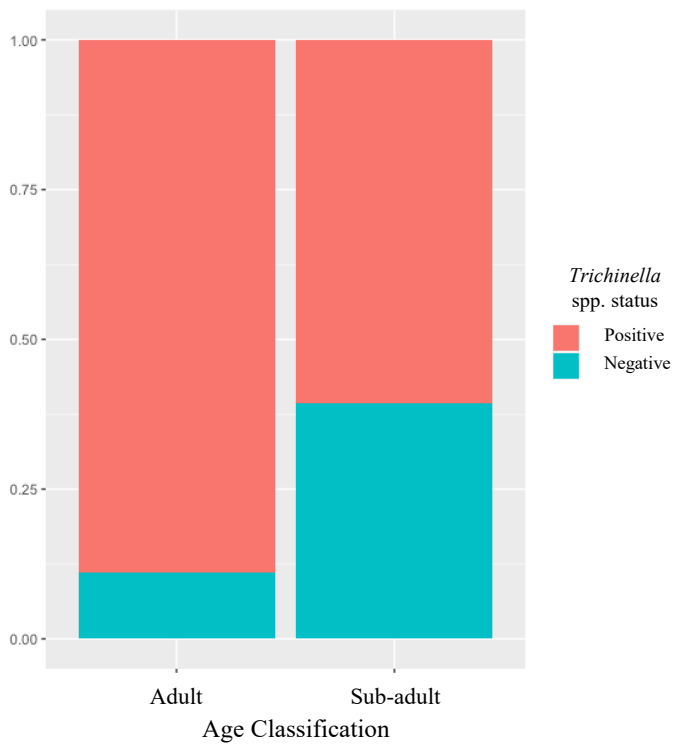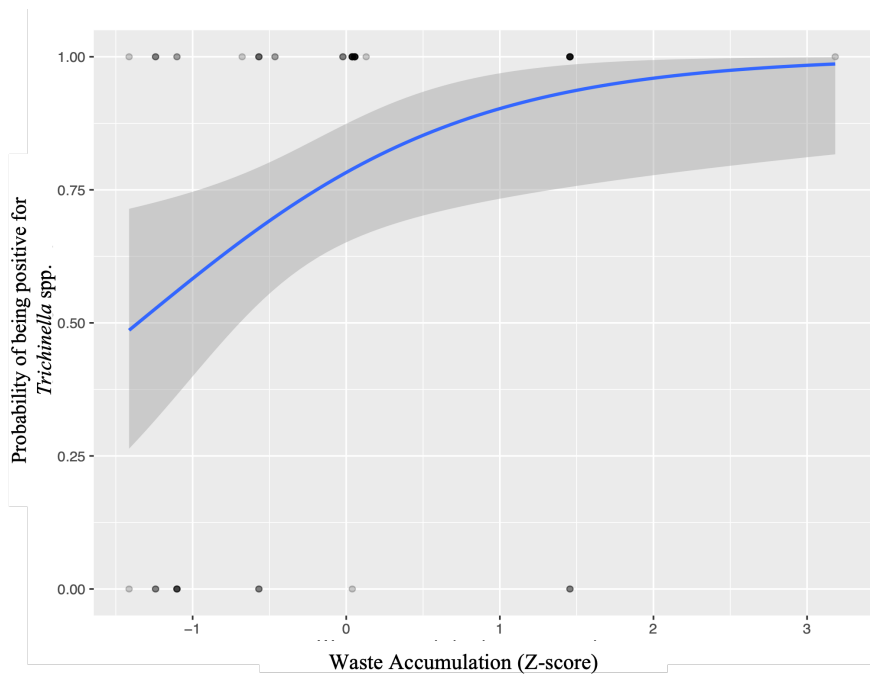

Supplement: Supplementary file 5 — Supplementary Information 5. [file 41598_2024_62800_MOESM5_ESM.pdf]
